# A 3D SVZonChip Model for In Vitro Mimicry of the Subventricular Zone Neural Stem Cell Niche

**DOI:** 10.3390/bioengineering12060562

**Published:** 2025-05-23

**Authors:** Ioannis Angelopoulos, Konstantinos Ioannidis, Konstantina Gr. Lyroni, Dimitris Vlassopoulos, Martina Samiotaki, Eleni Pavlidou, Xanthippi Chatzistavrou, Ioannis Papantoniou, Konstantinos Papageorgiou, Spyridon K. Kritas, Ioannis Grivas

**Affiliations:** 1 Laboratory of Microbiology and Infectious Diseases, School of Veterinary Medicine, Faculty of Health Sciences, Aristotle University of Thessaloniki, University Campus, 54124 Thessaloniki, Greece; kvpapageorgiou@vet.auth.gr (K.P.); skritas@vet.auth.gr (S.K.K.); 2 Skeletal Biology and Engineering Research Centre, Department of Development and Regeneration, KU Leuven, O&N1, Herestraat 49, PB 813, 3000 Leuven, Belgium; konstantinos.ioannidis@kuleuven.be (K.I.); ioannis.papantoniou@kuleuven.be (I.P.); 3 Prometheus Division of Skeletal Tissue Engineering, KU Leuven, O&N1, Herestraat 49, PB 813, 3000 Leuven, Belgium; 4 Foundation for Research and Technology (FORTH), Institute for Electronic Structure and Laser, 70013 Heraklion, Greece; klyroni@materials.uoc.gr (K.G.L.); dvlasso@iesl.forth.gr (D.V.); 5 Department of Materials Science and Engineering, University of Crete, 70013 Heraklion, Greece; 6 Biomedical Sciences Research Center “Alexander Fleming”, 16672 Vari, Greece; samiotaki@fleming.gr; 7 School of Physics, Faculty of Sciences, Aristotle University of Thessaloniki, 54124 Thessaloniki, Greece; elpavlid@auth.gr; 8 Department of Chemical Engineering, Aristotle University of Thessaloniki, 54124 Thessaloniki, Greece; xchatzist@cheng.auth.gr; 9 Laboratory of Anatomy, Histology and Embryology Veterinary School, Faculty of Health Sciences, Aristotle University of Thessaloniki, 54124 Thessaloniki, Greece; janos@vet.auth.gr

**Keywords:** subventricular zone (SVZ), neural stem cells (NSCs), 3D organotypic in vitro culture, organ on a chip (OCC), microfluidics, biofabrication, tissue engineering

## Abstract

Neural stem cells (NSCs) are crucial components of the nervous system, primarily located in the subventricular zone (SVZ) and subgranular zone (SGZ). The SVZ neural stem cell niche (NSCN) is a specialized microenvironment where growth factors and extracellular matrix (ECM) components collaborate to regulate NSC self-renewal and differentiation. Despite its importance, our understanding of the SVZ remains incomplete due to the inherent challenges of animal research, particularly given the tissue’s dynamic nature. To address these limitations, we developed a proof-of-concept, dynamic, and tissue-specific 3D organotypic SVZ model to reduce reliance on animal models. This static 3D organotypic model integrates a region-specific decellularized ECM derived from the SVZ, mimicking the native NSCN and supporting mouse-derived ependymal cells (ECs), radial glial cells (RGCs), astrocytes, and NSCs. To further improve physiological relevance, we incorporated a dynamic microfluidic culture system (SVZonChip), replicating cerebrospinal fluid (CSF) flow as observed in vivo. The resulting SVZonChip platform, combining region-specific ECM proteins with dynamic culture conditions, provides a sustainable and reproducible tool to minimize animal model use. It holds significant promise for studying SVZ-related diseases, such as congenital hydrocephalus, stroke, and post-stroke neurogenesis, while advancing translational research and enabling personalized medicine protocols.

## 1. Introduction

Neurogenesis in the human cortex begins with neuroepithelial stem cells (NESCs), which divide symmetrically to create radial glial cells (RGCs). These RGCs then divide asymmetrically to produce nascent projection neurons and additional RGCs [1]. Following neurogenesis, the radial scaffold of RGCs detaches from the apical surface and generates astrocytes and ependymal cells (ECs) through asymmetric divisions. In the adult brain, neural stem cells (NSCs) reside in specialized microenvironments known as NSC niches (NSCNs) [2]. The primary adult NSCN includes the ventricular-subventricular zone (V-SVZ) of the lateral ventricles and the subgranular zone (SGZ) of the dentate gyrus [3]. This study focuses exclusively on the SVZ NSCN. The mammalian SVZ comprises four distinct layers: the ependymal layer, hypocellular layer, astrocytic ribbon, and transitional layer [4]. It serves as the primary region for new NSC production. Within the SVZ, NSCs (B1 cells) exhibit astroglial characteristics (SOX2+, Nestin+, GFAP+, and Ki67+) and give rise to intermediate B2 cells (Nestin+, Vimentin+, and GLAST+), which lose apical contact with the cerebrospinal fluid (CSF) [5,6]. ECs (marked by acetylated α-tubulin, γ-tubulin, Pericentrin, and S100β) surround the B1 cells, forming pinwheel structures essential for neurogenesis regulation [6]. A detailed overview of these cell populations can be found in Del Bigio [7]. The SVZ architecture is heavily influenced by the extracellular matrix (ECM), which changes its composition during development [8]. The ECM consists of three layers: the basement membrane (laminin, fibronectin, and heparan sulfate), perineuronal nets (hyaluronate, proteoglycans, tenascin R, and link proteins), and the neural interstitial matrix. The ECM not only provides structural support but also regulates neural development, as disruptions in its organization are linked to cortical malformations and neurodevelopmental disorders [9]. Transcription factors such as Notch, Neurog2, Tbr2, Prox1, NFIX, Tlx, CcnD2, and Ascl1 further influence NSC behavior [10]. Additionally, ECM glycosaminoglycans and proteoglycans have been shown to modulate NSC self-renewal and differentiation [8]. The apical surface of the SVZ, which is exposed to the CSF, plays a key role in regulating neurogenesis [11]. Mechanical forces from the CSF, including hydrostatic pressure, are known to promote the proliferation of neuroepithelial progenitors [12,13,14]. Collectively, the interplay between cellular, chemical, and mechanical components within the SVZ NSCN is essential for maintaining the balance between NSC self-renewal and differentiation. To study the SVZ and its influence on NSCs, it is crucial to develop in vitro models that closely mimic the native microenvironment. Early models focused on co-culturing astrocytes with endothelial cells to replicate the blood–brain barrier (BBB) [15,16]. These platforms have enabled studies on pharmacological permeability and pathophysiology, as well as advancements in regenerative medicine (RM) [17]. However, traditional in vitro models, including transwell membranes [18,19,20], are limited by static fluid conditions, leading to unstable biochemical gradients and poor replication of in vivo SVZ dynamics [21]. Microfluidic platforms, such as organ-on-a-chip (OOC) systems, provide an opportunity to overcome these challenges [22,23,24]. By simulating multi-organ interactions, these platforms enable pharmacology, physiology, and toxicology studies [25,26,27] with reduced reliance on animal models, aligning with the 3Rs principle (replacement, reduction, and refinement) [28,29,30,31,32]. Notable advantages of microfluidics include reduced sample volumes, cost efficiency, and rapid results [33,34,35,36]. Proof-of-concept BBB models have demonstrated the potential of OOC platforms for studying complex physiological properties and advancing drug development [37,38,39]. However, dynamic SVZ models remain underdeveloped, limiting the ability to accurately replicate the in vivo SVZ NSCN. A decellularized extracellular matrix (dECM) has emerged as a promising tool for tissue engineering due to its ability to mimic native cellular signals and biochemical microenvironments [40,41,42,43,44,45,46]. Incorporating a region-specific ECM from SVZ tissue (svzECM) can advance in vitro models by recapitulating the chemical, structural, and mechanical properties of the native SVZ microenvironment. Additionally, the role of CSF flow, which exerts shear stress on ECs, is critical for autoregulation in the SVZ NSCN [47]. To address current limitations, we developed a 3D organotypic SVZ-on-a-chip model (SVZonChip) that incorporates ECs and RGCs derived from the mouse SVZ. These cells were encapsulated in a bovine svzECM hydrogel within a microfluidic device connected to a peristaltic pump to mimic CSF flow. This platform replicates SVZ NSCN physiology, including structural integrity and permeability, while enabling dynamic culture conditions. By integrating microfluidic technology with a region-specific ECM, the SVZonChip offers a novel approach for studying diseases such as congenital hydrocephalus, stroke, post-stroke SVZ neurogenesis [48], and SVZ-originating glioblastoma [49].

## 2. Materials and Methods

### 2.1. Decellularization of the Bovine SVZ

The SVZ (svzECM) was decellularized according to the protocol presented by Zheng Y et al. [50]. The bovine brain was provided by a local slaughterhouse in the Macedonia region (Farma Chalastras S.A) in Greece. The removal of the brain (craniotomy), as well as the excision of the SVZ, was performed by the veterinarian responsible for the unit. The brain was cut in half using a surgical scalpel, and the SVZ was then isolated. In total, two SVZs were removed from the lateral ventricles of the bovine brain. After isolating the SVZ, the tissue was rinsed with phosphate-buffered saline (PBS) (Gibco, Grand Island, NY, USA) and cut into 1 cm^3^ pieces. Later, the tissue was soaked in 1% sodium dodecyl sulfate (SDS) for 5 days with continuous stirring. The tissue sections were rinsed in water for 5 days, with the water being refreshed twice a day, then freeze-dried (Coolsafe^TM^, Scanvac, Wrocław, Poland) and stored at −20 °C.

### 2.2. Characterization and Relative Quantification of the Protein Composition of the Bovine svzECM Decellularized Tissue

The protocol for liquid chromatography–mass spectrometry (LC–MS) was conducted using a previous protocol defined by Bekas N. et al. [51]. Specifically, the svzECM tissue protein extract underwent tryptic digestion following the Sp3 protocol, which included an alkylation step with 100 mM iodoacetamide (Thermo Scientific Chemicals, Ward Hill, MA, USA). Later, 20 μg of beads (Cytiva Life Sciences, Marlborough, MA, USA) were added to each sample in 50% ethanol. The proteins were then cleaned using a magnetic rack. The beads were washed twice with 80% ethanol and once with 100% acetonitrile (Fisher Scientific, Waltham, MA, USA). The proteins bound to the beads were digested overnight at 37 °C with vigorous shaking (Eppendorf, Hamburg, Germany), using 1 μg of trypsin/LysC (MS grade, Promega, Madison, WI, USA) in 25 mM ammonium bicarbonate. The following day, the peptides were purified using a modified Sp3 clean-up protocol, then solubilized in mobile phase A (0.1% formic acid in water) and sonicated. The peptide concentration was measured at an absorbance of 280 nm with a NanoPhotometer^®^ P330 (Implen, Village, CA, USA). The samples were analyzed using an LC–MS system, which included a Dionex Ultimate 3000 nanoRSLC (Thermo-Fisher Scientific, USA) connected in-line with a Thermo Q Exactive HF-X Orbitrap mass spectrometer (Thermo-Fisher Scientific, USA). Peptide samples were directly injected and separated on a 25 cm long analytical C18 column (PepSep, Marslev, Denmark, 1.9 μm beads, 75 μm ID) using a one-hour run. The gradient started with 7% Buffer B (0.1% formic acid in 80% acetonitrile) and increased up to 35% over 40 min, followed by a rise to 45% in 5 min, a further increase to 99% in 0.5 min, and then maintenance at that level for 14.5 min to allow for equilibration. A full MS scan was performed in profile mode on a Q Exactive HF-X Hybrid Quadrupole-Orbitrap mass spectrometer, covering a scan range of 375–1400 *m*/*z* with a resolution of 120 K, an AGC target of 3 × 10^6^, and a maximum IT of 60 ms. This was followed by data-independent acquisition using 8 Th windows (a total of 39 loop counts), each with a resolution of 15 K, an AGC target of 3 × 10^5^, a maximum IT of 22 ms, and a normalized collision energy (NCE) of 26. The raw data from the Orbitrap were analyzed using DIA-NN (data-independent acquisition by neural networks) [52] against the bovine proteome (downloaded from Uniprot). The analysis was conducted in the software’s library-free mode (version 1.8.1), permitting up to two missed tryptic cleavages and a maximum of three variable modifications per peptide. A spectral library was generated from the DIA runs and then used to reanalyze them in double search mode. A DIA-NN search was conducted with oxidation of methionine residues, N-terminal methionine excision, and acetylation of protein N-termini specified as variable modifications, while carbamidomethylation of cysteine residues was set as a fixed modification. The match-between-runs feature was applied for all analyses, with the output (precursor) filtered at a 0.01 FDR. Protein inference was conducted at the gene level, utilizing only proteotypic peptides.

### 2.3. Fabrication of the Bovine svzECM Hydrogel

The svzECM hydrogel fabrication process is described in detail in the following paragraph. The lyophilized svzECM was homogenized and then further digested at 1 mg/mL in 0.01 M HCl using 1 mg/mL pepsin (Sigma, St. Louis, MO, USA), which was stirred at a constant rate using a magnetic stirrer (Vevor, Rancho Cucamonga, CA, USA) for 3 consecutive days at room temperature. The sample was divided into aliquots and stored at −20 °C for long-term preservation. The hydrogel-derived svzECM (1 mg/mL) was fabricated with the resulting digestive solution and neutralized using the following solutions: collagen type I (Thermofisher) in a ratio of 80/20 (collagen type I/svzECM), 10Χ PBS (Gibco), NaOH (1 M) (Sigma), and DMEM culture medium (Gibco) to prepare an svzECM pre-hydrogel. The hydrogel mixtures were prepared on ice by combining the stock gel solutions with neutralizing diluents to achieve the desired concentrations. The liquid pre-hydrogel was left at 37 °C for about 30 min until gelation occurred, showing a semisolid hydrogel.

### 2.4. Characterization of the Bovine svzECM Hydrogel

The svzECM hydrogel samples were prepared according to Section 2.3, stored at −20 °C, and lyophilized. Later, the samples were sublimated in a vacuum for carbon coating. This process was performed with a JEOL-4X vacuum sublimator, and the thickness of the carbon coating did not exceed 200 Å. This procedure was performed before the observation and elemental analysis of the samples with scanning electron microscopy supplemented with an X-ray energy dispersive spectrometer (SEM/EDS) (JEOL JSM-IT300, Akishima, Japan). The imaging of the samples was performed with a FESEM JSM 7610 F PLUS (field emission scanning electron microscope) by JEOL with integrated EDS and the analytical system AZTEC by OXFORD. The measurements were carried out with a secondary electrons (SEI) detection system, with a voltage of 5–15 kv and at a working distance of approximately 8 (WD 8). In addition, the magnification and the corresponding scale were indicated in each image.

### 2.5. Rheological Characterization of the Bovine svzECM Hydrogel

To obtain the flow characteristics of the svzECM hydrogel, rheological measurements were performed. The svzECM hydrogel was prepared as described in Section 2.1, and then its rheological properties were measured with an Anton Paar MCR-502 rheometer, using a cone plate (CP) geometry (with a stainless steel top cone and a bronze bottom plate), a diameter of 25 mm, truncation of 50 μm, and an angle of 0.3 rad. The svzECM hydrogel was gently placed on the bottom plate, taking care to avoid bubble formation. This plate was roughened mechanically (typical roughness was about 0.3 mm) to reduce the risk of wall slip. A 100 cp PDMS sealing fluid was used around the edges of the hydrogel to mitigate ege effects (mainly evaporation). After the sample was placed on the bottom plate, the top conical geometry was gradually lowered until it reached a distance of 50 μm from the properly aligned bottom plate (truncation). A dynamic frequency sweep test was conducted at 37 °C (controlled with a Peltier element), with a strain amplitude of 0.5% in the linear regime over a frequency range 10^2^–10^−2^ rad/s. Subsequently, a dynamic strain sweep test from 10^−2^ to 10^2^% was carried out at a constant angular frequency of 1 rad/s, followed by a dynamic time sweep at 1 rad/s and a strain amplitude of 0.5%.

### 2.6. Mouse SVZ Isolation and Postnatal RGC Cell Culture

The mice were housed in the animal facility at the Veterinary School of the Aristotle University of Thessaloniki. All animal experiments were approved by the Veterinary Administration of the Prefecture of Macedonia, Greece, and were carried out in strict compliance with EU Directives (reference number: 870027/3584). All animal procedures followed a protocol approved by the Institutional Animal Care and Use Committee of the Aristotle University of Thessaloniki (reference number: 22954/2024) and followed the In Vivo Experiments (ARRIVE) guidelines/checklist for preclinical animal studies. The mouse SVZ was extracted from postnatal (P0–P3) mice using a surgical procedure detailed in previous descriptions [53,54]. Specifically, the brain was extracted, and the lateral ventricle was dissected from the caudal region of the telencephalon, with the hippocampus and septum removed. For in vitro cultures of postnatal RGCs (pRGCs), the walls of the lateral ventricles from postnatal mice were dissected, mechanically dissociated, and plated on poly-D-lysine-coated (10 μg/mL, Thermofisher) plates (Nunc, Thermofisher). The pRGCs were cultured in a proliferation medium composed of DMEM-high glucose (Gibco), 10% fetal bovine serum (FBS) (Gibco), 1% penicillin/streptomycin (Gibco), epidermal growth factor (EGF), and basic fibroblast growth factor (bFGF) (Peprotech, Cranbury, NJ, USA) to maintain their progenitor phenotype, whereas, to differentiate pRGCs towards ECs, the medium was changed to a differentiation medium consisting of DMEM-high glucose (Gibco), 2% FBS (Gibco), and 1% penicillin/streptomycin (Gibco) and maintained for 15 days under continuous incubation according to previous protocols [53,55].

### 2.7. Cell Proliferation Assay

To evaluate the proliferation capacity of pRGCs, 500,000 cells were encapsulated in an svzECM hydrogel, as outlined in Section 2.3. The cells were cultured in a proliferation medium containing DMEM-high glucose (Gibco), 10% FBS (Gibco), and 1% penicillin/streptomycin (Gibco) and were maintained at 37 °C with 5% CO_2_ to preserve their progenitor phenotype. Proliferation rates were assessed on days 1 and 5 using the MTT proliferation assay, following the manufacturer’s instructions (Roche). Absorbance readings were obtained using a plate reader (Tecan, Chapel Hill, NC, USA) at 450 nm, with 570 nm as the reference wavelength.

### 2.8. Static and Dynamic 3D Organotypic Models

The fabrication of either the static or the dynamic 3D organotypic model (n = 2 pups for each 3D organotypic model) was assembled inside a class II cabinet. A timeline of the different phases of the development of the 3D organotypic models (static or dynamic) was constructed to guide the experimental process. For the static 3D organotypic model, the polycarbonate (PCF) membranes of the inserts (Millicell-12 mm insert, 0.4 μm, Millipore, Billerica, MA, USA) were coated with poly-D-lysine (10 μg/mL, Thermofisher) for 1 h at room temperature, rinsed with double-distilled water (ddH_2_O), and then dried for 1 h in a sterile hood at room temperature. Specifically, a 100 µL drop of 500,000 pRGCs was cultured at the lower part of the insert (on top of the membrane) and left to attach to the membrane overnight at 37 °C and 5% CO_2_. After 24 h, another drop of 500,000 pRGCs was added, and the cells were cultured at the upper part of the insert (on top of the membrane) and left at 37 °C and 5% CO_2_. Then, 200 μL of the proliferation medium was added to the upper chamber (and replaced every other day), and 200 μL of the differentiation medium was added to the lower chamber. The two transwell chambers had the same culture medium during the first two days of culture. Then, the medium in the lower chamber was changed to the differentiation medium (DMEM-2% FBS-1% P/S) for 15 consecutive days, as described in Section 2.6. In parallel, for the dynamic 3D organotypic model, the microfluidic device (Quasi Vivo^®^ QV600, Kirkstall Ltd., York, UK) was first sterilized with 70% ethanol. Later, the solvent was discarded, the components were dried, and PBS was flushed through the system to eliminate any remaining traces of ethanol. The PCF membrane inserts (Millicell-12 mm insert, 0.4 μm, Millipore) were prepared as described above. Specifically, a 100 µL drop with 500,000 pRGC cells was cultured at the lower part of the insert (on top of the membrane) and left to attach well to the membrane overnight at 37 °C and 5% CO_2_. After 24 h, the svzECM hydrogel was prepared as described in Section 2.3, containing 500,000 pRGGs and cultured at 37 °C and 5% CO_2_ to allow the cross-linking process to occur. Then, the insert containing the 3D organotypic model was installed in the microfluidic device. Three chambers were configured within the system to enable the simultaneous operation of the three 3D organotypic models under identical experimental conditions. An O-ring was used to prevent the leakage of the medium between the upper and the lower chambers. The microfluidic device was connected to a reservoir bottle (20 mL) containing the differentiation medium (as described in Section 2.6), which was running only in the lower chamber, with an air filter (0.2 μm PES; Corning, Corning, NY, USA), two inlet tubes (1/16“), pump tubing, and one outlet tube (3/32“). Later, 400 μL of the proliferation medium (as described in Section 2.6) was added to the upper chamber carefully without dislodging the svzECM hydrogel. Finally, the microfluidic system was placed inside a cell culture incubator and connected to a one-channel peristaltic pump (Quasi Vivo QVMP1) to produce a pulsatile, laminar, and unidirectional flow of differentiation medium at a constant flow rate (0.1 µL/min) and left at 37 °C and 5% CO_2_ for 15 consecutive days. In the upper chamber, a static culture was maintained by changing the proliferation medium every other day; however, in the lower chamber, the differentiation medium was continuously recycled for the period of 15 days to differentiate the pRGCs into ECs. The development of the 3D organotypic model was structured into distinct phases to facilitate experimental reproducibility.

### 2.9. Immunocytochemistry

The expression of progenitor pRGC and EC proteins was analyzed via immunocytochemistry using monoclonal antibodies specific to mouse anti-acetylated α-tubulin (acTub) (1:400, Santa Cruz, CA, USA), rabbit anti-Foxj1 (1:1500, Sigma), mouse anti-Foxj1 (1:1500, eBioscience), and rabbit anti-p73 (Abcam, Cambridge, UK). pRGCs were cultured in 35 mm FluoroDish™ cell culture dishes (World Precision Instruments, Sarasota, FL, USA) in a proliferation medium to maintain their progenitor phenotype (as described in Section 2.6). To induce differentiation into ECs, the medium was replaced with a differentiation medium, and the cells were incubated continuously for 15 days (as described in Section 2.6). On the day of the response, the medium was removed, and the cells were fixed with 4% formaldehyde in PBS for 10 min at room temperature. After PBS washes, cells were blocked for nonspecific binding and permeabilized with 3% BSA and 0.1% Triton X-100 in PBS for 1 h at room temperature. Primary antibodies diluted in PBS were applied to each dish and incubated overnight at 4 °C. The cells were then washed and incubated with secondary antibodies conjugated to AlexaFluor 488 (donkey anti-mouse, Santa Cruz, CA, USA) or AlexaFluor 594 (donkey anti-rabbit, Santa Cruz, CA, USA) at a 1:200 dilution for 1 h at room temperature. DNA was counterstained with DAPI (1:1500, Sigma) or Hoechst staining (1:1500, Sigma), and the samples were mounted with glass coverslips using the ProLong antifade mounting medium (Fisher Scientific). Images were acquired using a Leica TCS SP5 (Wetzlar, Germany) confocal fluorescence microscope equipped with a Leica DMI6000B system and 40× and 63× objectives.

### 2.10. Histology and Immunohistochemistry

For hematoxylin–eosin (H&E) (Merck, Darmstadt, Germany) staining, adult mouse brains, the 3D organotypic model, and the decellularized svzECM were fixed with 10% formalin (Sigma) and then embedded in paraffin (Sigma). Slices of 10 μm thick sections were mounted on positively charged glass slides. After deparaffinization with xylene, the samples were dehydrated using a graded alcohol series (50%, 70%, 90%, and 100% alcohol) and finally stained with H&E. Similarly, for immunofluorescence, mouse brains and the 3D organotypic model were fixed with 4% PFA and subsequently washed with PBS. Wholemount samples were treated with 0.3% Triton X-100 for 10 min and incubated in a blocking solution containing 10% FBS, 3% BSA, and 0.1% Tween 20 in 1× PBS for 1 h. Then, they were incubated for 1 h at room temperature in a blocking solution containing 10% normal goat serum in 0.1 M PBS with Triton-X100. Samples were incubated overnight at 4 °C with primary antibodies in a blocking solution. The primary antibodies used were the following: (1) rabbit anti-GFAP (1:400, DakoCytomation, Glostrup, Denmark), (2) mouse anti-acetylated α tubulin (acTub) (1:400) (Santa Cruz, CA, USA), (3) rabbit anti-β-catenin (1:500) (Sigma), (4) rabbit anti-Sox2 (1:500) (Abcam), and (5) mouse anti-Sox2 (1:500) (Abcam). After incubating the samples overnight at 4 °C in a humid chamber, they were washed with PBS (Gibco) and then incubated with a secondary antibody conjugated to AlexaFluor 488 (donkey anti-mouse, Santa Cruz, CA, USA) or AlexaFluor 594 (donkey anti-rabbit, Santa Cruz, CA, USA) at a concentration of 1:200. Finally, DNA was stained with DAPI (1:1500, Sigma) or Hoechst staining (1:1500, Sigma), and the samples were mounted with Prolong antifade (Fisher Scientific). H&E images were taken with a Nikon Eclipse TE2000-U microscope and collected using a Nikon Digital Sight DS-L1 camera. Images from immunohistochemistry were captured using a Leica TCS SP5 confocal fluorescence microscope with a Leica DMI6000B microscope and 40× and 63× lenses. Digital images were processed using Adobe Photoshop (version 26.6.1) (San Jose, CA, USA) and Fiji software (version Fiji 2.14.0) University of Wisconsin–Madison (USA). For 3D morphological analysis, raw z-stack images were processed with Fiji, and 3D reconstructed snapshots were generated using Imaris 9.1 (Bitplane, UK). The area of the acTub+ cilia signal over the total area was quantified from confocal images using ImageJ University of Wisconsin–Madison (USA, https://imagej.net/ij/). Briefly, wholemount images of the native mouse SVZ, along with the static and dynamic organotypic 3D models, were used to isolate the z-stacks containing only cilia, which were subsequently overlaid. The image type was then converted to RGB, and a colored threshold was applied for the selection and quantification of the area corresponding to acetylated-cilia-positive (acTub+) signals. For quantification, three images per sample were analyzed, with three regions of interest (ROIs) selected per image to calculate the average signal intensity.

### 2.11. Statistical Analysis

All experiments were conducted in biological and experimental triplicate (n = 3), with results presented as the mean. Statistical analyses were performed using GraphPad Prism software version 7. Group comparisons were carried out using *t*-test or one-way ANOVA followed by post hoc Tukey. Statistical significance was determined at *p* < 0.05 (*), *p* ≤ 0.01 (**), and *p* ≤ 0.001 (***).

## 3. Results

### 3.1. Bovine SVZ Decellularization

**Figure 1:**
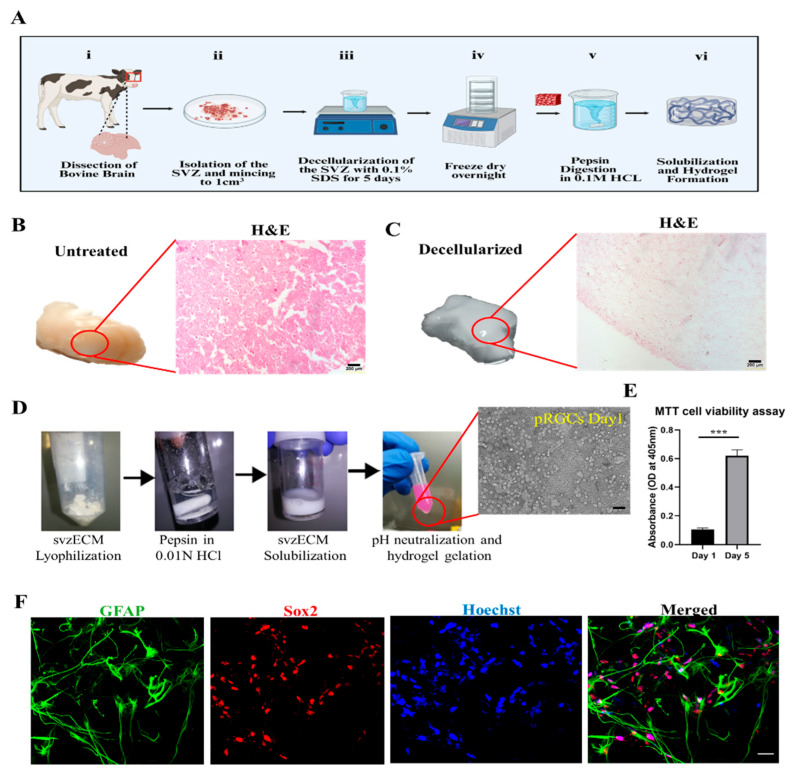
Decellularization of the bovine SVZ tissue. (**A**) Schematic illustration showing the decellularization process we followed. (**B**) Diced bovine SVZ tissue prior to the decellularization process. The tissue has a distinct brownish color, which is also confirmed by the presence of cells after H&E staining. (**C**) Immersing bovine SVZ tissue in a 1% SDS solution leads to cell lysis and the elimination of cellular components. After 7 days, the tissue starts to lose its color, indicating the removal of cellular material. Ultimately, the tissue becomes fully blanched, leaving only the extracellular matrix. H&E staining shows the almost complete removal of cellular material while maintaining the integrity of the svzECM structure. (**D**) Decellularization steps: (i) svzECM lyophilization, (ii) preparation of the pepsin solution in 0.01 N HCl, (iii) solubilization of the svzECM in pepsin solution, and (iv) pH neutralization and hydrogel gelation after the encapsulation of pRGCs. (**E**) MTT cell viability assay performed on pRGCs on day 1 and day 5 in the proliferation medium, n = 3, *p* ≤ 0.001 (***). (**F**) Immunofluorescent images of pRGCs after 5 days in the svzECM hydrogel, stained against GFAP (pRGCs marker), Sox2 (Stemness marker), and cell nuclei with Hoechst. Scalebars: (**B**,**C**): 200 μm, (**D**): 20 μm, (**F**): 50 μm. Created in BioRender (https://www.biorender.com/). Angelopoulos, I. (2025) https://BioRender.com/f28a555 (accessed on 15 September 2024).

Bovine brain tissue from a regional slaughterhouse was used for the decellularization of the SVZ to increase the svzECM yield and to minimize the use of experimental animals and adhere to the 3Rs, following European Union rules on the minimization of experimental animals in research. The protocol that we followed for the development of an SVZ region-specific hydrogel can be seen in the schematic illustration in Figure 1A. The untreated brownish native tissue of the SVZ can be seen in Figure 1B, along with hematoxylin and eosin (H&E) staining, showcasing that the organ’s microarchitecture was macroscopically intact. After 5 days of decellularization and SDS perfusion, the tissue became whitish and non-translucent, with a degree of ductility. Histological analysis was performed to assess the success of decellularization by examining the absence of cellular material and the preservation of the ECM’s microstructure. H&E staining revealed the removal of cellular material in the decellularized svzECM, while the matrix and its architecture remained intact without any significant damage (Figure 1C). Overall, the histological analyses confirmed successful decellularization, with the removal of most cellular components while the ECM structure was largely maintained. The basement membrane architecture was well-preserved after decellularization, despite the clear removal of nucleic material, indicated by the absence of hematoxylin nucleic counterstaining. In Figure 1D, the decellularization steps can be observed as follows. Initially, after dicing the bovine SVZ, the svzECM was lyophilized overnight prior to the preparation of the pepsin solution in 0.01 N HCl. Subsequently, the solubilization of svzECM took place in the pepsin solution and was stored at −20 °C until further use. Prior to seeding the svzECM with cells, the svzECM was thawed, and the pH was neutralized using 0.3 M NaOH to allow hydrogel gelation after encapsulating the pRGCs. The MTT assay results demonstrated that the encapsulated pRGCs were proliferating after 5 days within the svzECM without hindering cell viability (*p* ≤ 0.001 (***)), as shown in Figure 1E. Immunofluorescent images of samples on day 7 revealed the presence of Sox2+ and GFAP+ cells within the bovine svzECM and showcased the 3D arrangement of the cells within the gel matrix, as shown in Figure 1F. While the cell viability and phenotype were satisfactory, the svzECM hydrogel deformed and lost the integrity of its shape after 7 days in static culture conditions. Considering the eventual goal of incorporating dynamic shear forces in a microfluidic system, we analyzed the svzECM’s composition and developed a strategy to enhance hydrogel stability for extended dynamic culture.

### 3.2. Quantitative Characterization of the Decellularized Bovine svzECM

**Figure 2:**
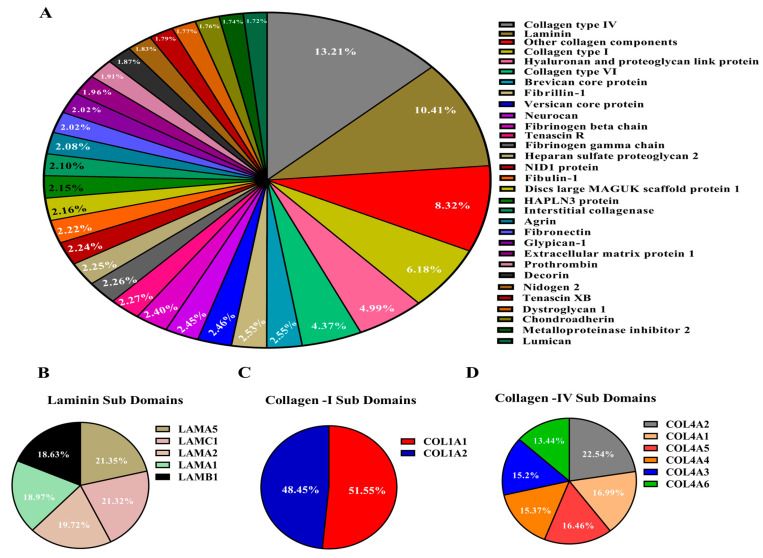
Mass spectroscopy of the decellularized svzECM tissue. Analysis of decellularized svzECM tissue to assess ECM protein composition (all ratios are reported as mass percentages). (**A**) Proteins related to the basal lamina, including structural proteins, were detected, with collagen IV and laminin being the most abundant. (**B**) Typical laminin isoforms, with LAMA5 and LAMC1 being more abundant. (**C**) Collagen I was also identified with COL1A1 as being more abundant, and (**D**) collagen IV A1 and A2 chains were more abundant compared to other isoforms.

In this study, the proteomic composition of decellularized bovine svzECM tissue was examined using the effective decellularization method described in Section 2.3. To gain in-depth insights into the svzECM proteins, a proteomic analysis of the decellularized bovine SVZ was carried out using LC–MS. The identified proteins were subsequently classified into various divisions of the svzECM using DIA-NN against the bovine proteome. The svzECM was divided into two main components: (1) the core matrisome, which includes collagens, proteoglycans, and glycoproteins, and (2) the ECM-associated division, comprising secreted factors, ECM regulators, and ECM-affiliated proteins. A total of 31 distinct ECM proteins were identified in the svzECM samples, which were further classified into core matrisome proteins and matrisome-associated proteins, as shown in Figure 2A. In the svzECM samples, a total of eight collagens and five laminins were identified, all belonging to the core matrisome. Additionally, ECM regulators, such as metalloproteinase inhibitor 2, and ECM-affiliated proteins, such as fibronectin, were identified in the matrisome-associated division of the svzECM samples. Collagens and laminins were the predominant components of the svzECM, with collagen IV being the most abundant, making up 13.2% of the total protein mass. Laminins were found to constitute 10.41% of the total protein mass, with subunits α5 (21.35%), γ1 (21.32%), α2 (19.72%), α1 (18.97%), and β1 (18.63%) detected, as shown in Figure 2B, indicating the preservation of these isoforms. Two types of collagen I were also identified, with α1 accounting for 48.45% and α2 for 51.55% of the collagen I proteins, as shown in Figure 2C. This is a relatively lower proportion than anticipated, possibly due to the average age of the bovine SVZ donors used for decellularization or the specific decellularization protocol employed. All six α chains of collagen IV were identified, with α1 and α2 being the most prevalent, accounting for 22.54% and 16.99% of the area, respectively. The other four subunits—α3, α4, α5, and α6—were found in proportions of 16.46%, 15.37%, 15.21%, and 13.44%, respectively. The decellularization process retained not only the COL4A1 and A2 chains, which are commonly found in all basement membranes, but also the COL4A3–A5 chains, as illustrated in Figure 2D. Several other ECM components were identified. Neurocan accounted for 2.45%, and tenascin R was detected at 2.27%, even with the use of a strong anionic detergent, as illustrated in Figure 2A. Nidogen-1, which acts as a connector between laminins and collagen IV in the formation of an interconnected basal lamina network, was detected at 1.83%. Other matrix components, such as fibronectin, which plays crucial roles in determining cellular fate and signaling [56], were found at 2.02%. To enhance the mechanical properties of the svzECM and extend its viability for prolonged dynamic cultures, we incorporated collagen type I into the final svzECM protocol. The solidified svzECM hydrogel was prepared at a concentration of 1 mg/mL through neutralization with collagen type I in an 80:20 ratio (collagen/svzECM) and supplemented with 10× PBS, NaOH, and DMEM medium. The mixture was then gelled at 37 °C for 30 min. The 80:20 collagen/svzECM formulation demonstrated superior structural integrity and was easier to handle compared to other ratios. This modification successfully reinforced the hydrogel, making it more stable and suitable for dynamic, long-term culture conditions.

### 3.3. Characterization of the Decellularized Bovine svzECM Hydrogel

**Figure 3:**
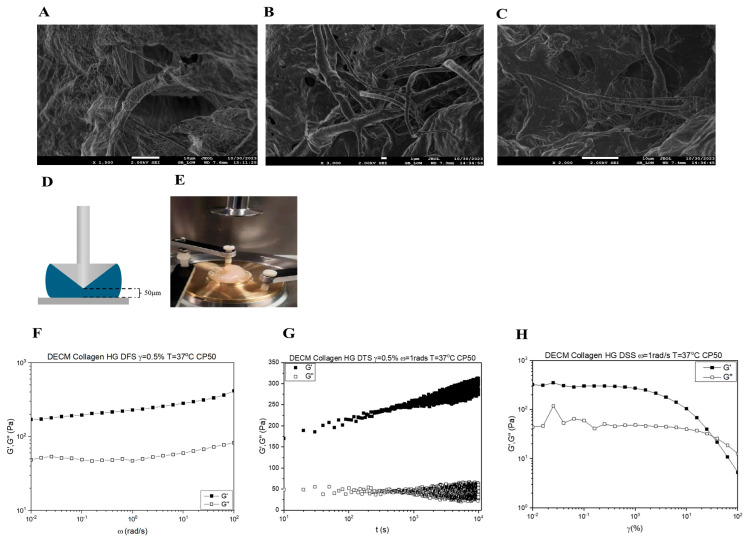
Characterization of the bovine decellularized svzECM hydrogel. (**A**–**C**) Scanning electron microscope (SEM) images of the svzECM hydrogel surface, scale bar: 1 μm. (**D**) Schematic showing the cone plate geometry used in the rheological study. The black dotted lines show the truncation, while the sample is shown in blue. (**E**) Photo of the Anton Paar MCR-501 rheometer, with the gold-colored base showing the specially designed brass base that has been added. The plastic cover is used to limit sample–air interactions. (**F**) Dynamic frequency sweep of the svzECM hydrogel. Similar behavior of a gel-like material is observed in DSS. (**G**) Dynamic strain sweep of the svzECM hydrogel with a constant angular frequency ω = 1 rad/s. The values of G′ are greater than G″, indicating that we have a viscoelastic solid. (**H**) Dynamic time sweep measurement of the svzECM hydrogel, where the sample was stable during the studied time.

The surface morphology of the reinforced svzECM was assessed using scanning electron microscopy (SEM). The SEM results of the svzECM gel also revealed a microstructure characterized by uniform and staggered collagen fibers. Large quantities of well-organized fibrils in collagen I gels were detected, as illustrated in Figure 3A–C. The differences in the microstructure likely reflect the amount of collagen I in each gel, since collagen IV, the most abundant protein in the svzECM, does not form fibrils. Collagen fibers in the svzECM hydrogel were observed, as well as areas where cells could adhere and grow. The rheological properties of the svzECM hydrogel, which consists predominantly of collagen and laminin, were investigated, with particular attention to the temporal stability of the material, its storage and loss moduli (G′ and G″, respectively), and modulus recovery following yielding and flow (emulating the self-healing process). As illustrated in Figure 3F, G′ is greater than G″ over the entire frequency range explored, indicating that the sample is a viscoelastic solid. In addition, the moduli exhibit very weak frequency dependence. This behavior is akin to a gel with an average plateau modulus of 250 Pa. This relatively low value is not uncommon for gels based on collagen or other networks based on long fibers. Figure 3G depicts the results of a dynamic strain sweep test. At low strains up to 1%, the moduli are virtually constant (linear viscoelastic regime), and the material exhibits solid behavior, consistent with the frequency spectrum in Figure 3F. With increasing strain, the material enters the nonlinear regime, which is characterized by a gradual decrease of both moduli and eventual yielding, i.e., the transition of the svzECM hydrogel from a viscoelastic solid to a viscoelastic liquid at a yield strain amplitude of γy = 30%. Τhe corresponding yield stress is σy ≈ 40 Pa, i.e., very weak. Hence, this gel made of collagen and other proteins is weak, deforms a little, and yields at low stress and low strain. Upon yielding and flow cessation, the hydrogel recovers its structure. This is indirectly, albeit convincingly, shown by the dynamic time sweep (at a strain amplitude of 0.5% and an angular frequency of 1 rad/s) in Figure 3H. There is a fast increase in the first 10 s (the modulus recovers about 58% of its original value at 175 Pa and then increases logarithmically, reaching 300 Pa at about 10^4^ s). Given the overall non-Brownian nature of collagen, this is an interesting result that is analogous to other classes of materials exhibiting logarithmic aging.

### 3.4. Developing a Static 3D Organotypic SVZ Model

**Figure 4:**
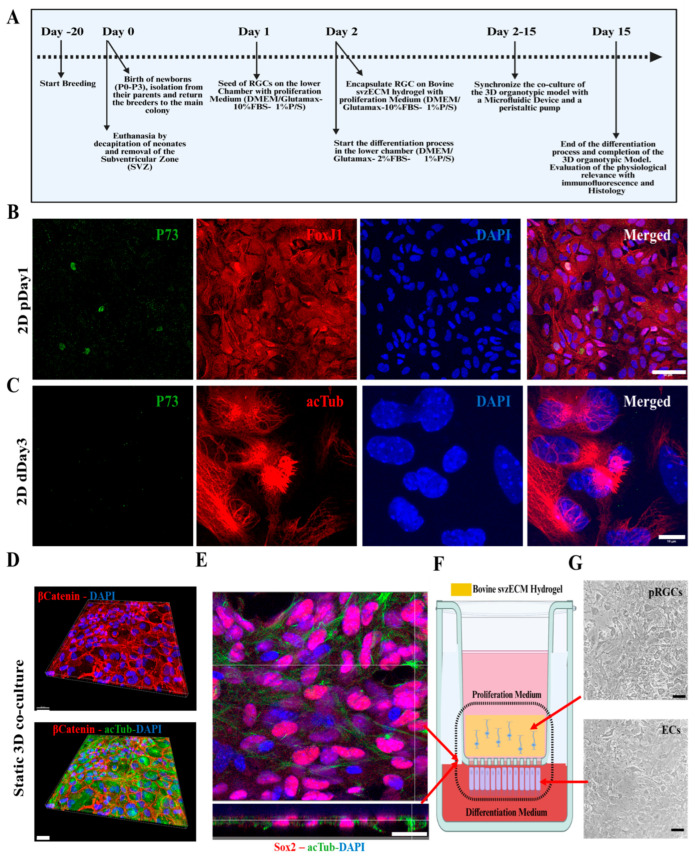
The phases of developing the 3D organotypic SVZ model. (**A**) Timeline of co-culture establishment: breeding starts 20 days before the establishment of the 3D organotypic model. The day of birth of pups (P0–P3) is considered day 0, where dissection of the later ventricles and the isolation and cell culture of pRGCs also occurred on the following days. On day 5 and day 6, the 3D organotypic model is formed, and the insert is placed inside the microfluidic device with the proliferation and differentiation mediums accordingly. Day 15 marks the end of the differentiation process of pRGCs into ependymal cells, and the mature formation of the 3D organotypic model. (**B**) Immunofluorescence images of 2D cultures of pRGCs maintained in the proliferation medium (DMEM-high glucose + 10% FBS + EGF + bFGF) for 1 day (“p”), prior to encapsulation in the svzECM, showing P73 (green), FoxJ1 (red), and DAPI (blue). (**C**) Immunofluorescence images of 2D cultures maintained in the differentiation medium (DMEM-high glucose + 2% FBS, no growth factors) for 3 days (“d”), showing acetylated tubulin (acTub, red), P73 (green), and DAPI (blue). As these transcriptional markers (P73 and FoxJ1) typically emerge later during ependymal differentiation, their low expression at this early timepoint is expected. In contrast, early signs of ciliogenesis can already be detected via acTub staining. (**D**) Development of the organotypic co-culture and wholemount images from the bottom of the membrane, showing the epithelial barrier created by β-catenin along with cilia generation, as shown by staining against acTub in green. (**E**) Immunofluorescent images showing the top and side views of the membranes of samples stained against Sox2, acTub, and DAPI. (**F**) Schematic showing the transwell insert and the co-culture environment of the static 3D co-culture after 7 days in vitro. Immunofluorescent images showing the top and side views of the membranes of samples stained against Sox2, acTub, and DAPI. (**G**) Brightfield images showing the top surface of the svzECM hydrogel, along with the bottom view of the membrane below. Scalebar: (**B**): 50 μm, (**C**): 10 μm, (**D**): 30 μm, (**E**): 50 μm, (**G**) BF: 20 μm. Created in BioRender. Angelopoulos, I. (2025) https://BioRender.com/n94l069 (accessed on 15 September 2024), Created in BioRender. Angelopoulos, I. (2025) https://BioRender.com/g30h237 (accessed on 15 September 2024).

The timeline of co-culture establishment is outlined in Figure 4A, beginning with the isolation and culture of pRGCs from neonatal mouse lateral ventricles (P0–P3). Primary pRGCs were initially seeded on the lower chamber of the PCF membrane insert and incubated overnight at 37 °C with the proliferation medium (as described in Section 2.6) to ensure adherence. After adhesion, the cell insert was inverted, and the medium was switched to differentiation conditions (aCSF) for three days. Subsequently, a new batch of pRGCs was isolated, encapsulated within the bovine svzECM, and placed in the upper chamber of the insert with the proliferation medium (as described in Section 2.6). Co-culture was initiated from this point, designated as day 1 of the static 3D organotypic co-culture. By day 15, differentiation into ependymal cells and maturation of the organotypic model were achieved. Immunofluorescent imaging demonstrated the phenotype of proliferating pRGCs prior to encapsulation, as marked by P73 and FoxJ1 (Figure 4B), and differentiation into ependymal cells by day 3, as confirmed by acTub and P73 staining (Figure 4C). To assess the early responsiveness of postnatal radial glia-like cells (pRGCs) to environmental cues, we cultured them for 1–3 days under either proliferation or differentiation conditions. As expected, p73 and FoxJ1 expression was minimal at these early timepoints, reflecting their undifferentiated state. In contrast, acetylated tubulin staining on day 3 revealed the onset of ciliogenesis in a subset of cells, marking an early morphological response to differentiation cues. Wholemount imaging revealed epithelial barrier formation, evidenced by β-Catenin, and cilia development, highlighted by acTub staining (Figure 4D) (Figure S1 and Video S1). A static co-culture environment was maintained with pRGCs in the upper svzECM compartment and ependymal cells forming an epithelial barrier on the membrane, as illustrated in Figure 4E (Figure S2 and Video S2). The structural organization of the system was confirmed through immunofluorescence and brightfield imaging, highlighting Sox2 expression and cilia formation on both the membrane and svzECM surface. The combination of the svzECM and the layered co-culture system effectively recapitulates key features of the SVZ microenvironment in vitro. For high-resolution images, please refer to the Supplementary Figures.

### 3.5. Developing a Dynamic 3D Organotypic SVZ Model by Integrating Uniaxial Microfluidic Flow

**Figure 5:**
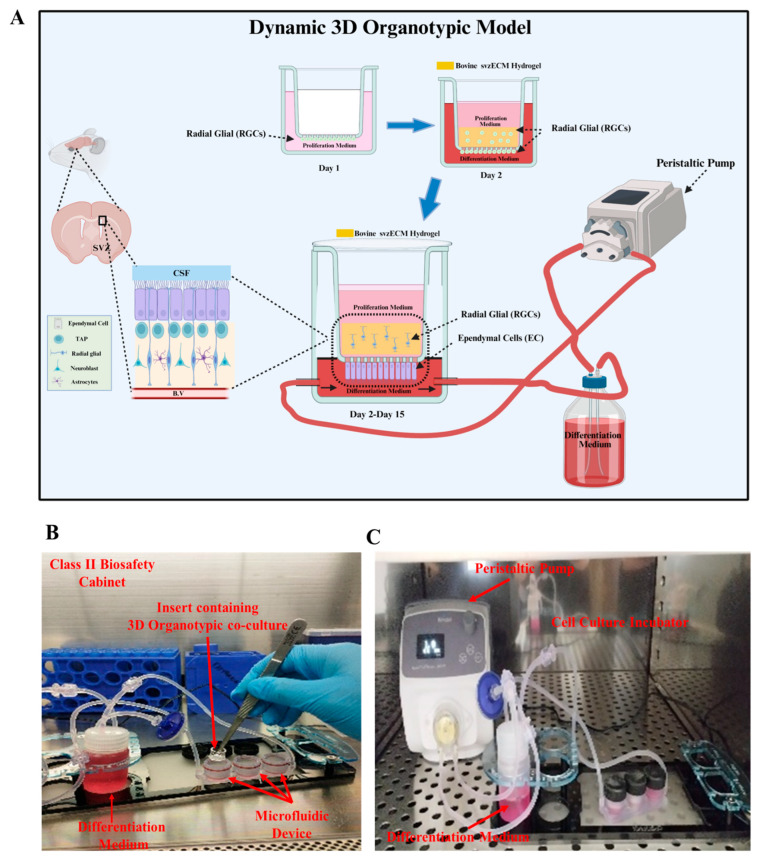
Dynamic 3D organotypic model for the SVZonChip. (**A**) Schematic of the dynamic 3D organotypic SVZ model. Radial glial cells (RGCs) were seeded on the PCF membrane insert in the proliferation medium (day 1) and overlaid with the bovine svzECM hydrogel containing encapsulated RGCs (day 2). The insert was connected to a peristaltic pump for continuous medium flow, mimicking the in vivo microenvironment and supporting RGC proliferation, differentiation, and epithelial barrier formation over 15 days. (**B**) Experimental setup showing the transwell insert within the microfluidic device in a Class II biosafety cabinet. (**C**) Peristaltic pump and incubator system maintaining a dynamic medium flow for long-term culture. Created in BioRender. Angelopoulos, I. (2025) https://BioRender.com/a55g039 (accessed on 15 September 2024).

To establish a dynamic 3D organotypic co-culture to mimic the SVZ, a microfluidic chamber from QuasiVivo had to be integrated with the already pre-established static setup by utilizing the transwell. The RGCs located on the lower chamber of the transwell were exposed to a differentiated medium with a constant dynamic flow of 0.1 μL/min for 15 days without causing dislodgment from the PCF membrane, enabling them to differentiate into ECs. The upper chamber contained the svzECM hydrogel with the RGCs in a proliferation medium under static conditions until confluency was reached. The svzECM hydrogel was designed to closely replicate the SVZ-specific extracellular matrix, creating a supportive environment for cellular structure and function in vitro. To mimic the in vivo gradient of factors, the co-culture system utilized a two-chamber setup separated by a permeable PCF membrane. The lower chamber contained differentiated pRGCs in a CSF medium, while the upper chamber housed encapsulated pRGCs within the svzECM hydrogel, cultured in a proliferation medium (as described in Section 2.6). This configuration allowed the diffusion of signaling molecules and nutrients across the membrane, establishing a gradient that facilitated communication between the two chambers, as shown in Figure 5B. This gradient effectively recreated the dynamic exchange of factors characteristic of the native SVZ microenvironment, supporting differentiation and epithelial barrier formation. The co-culture was maintained for 15 days continuously to achieve a 3D organotypic model that mimics the SVZ microenvironment. Additionally, the stability and functionality of the proposed SVZonChip were evaluated. There was no leakage of the culture medium from the upper chamber to the lower chamber, and vice versa, enabling continuous co-culture for up to 15 days. An O-ring surrounded the insert and was crucial for the experiment, preventing leakage from the upper chamber to the lower chamber and ensuring that the differentiation process was not interfered with. The constant flow of differentiation medium in the lower chamber mimicked the flow of the CSF, imitating the in vivo analog, as shown in Figure 5C.

### 3.6. Characterization of the Dynamic SVZonChip and Comparison with the Mouse SVZ

**Figure 6:**
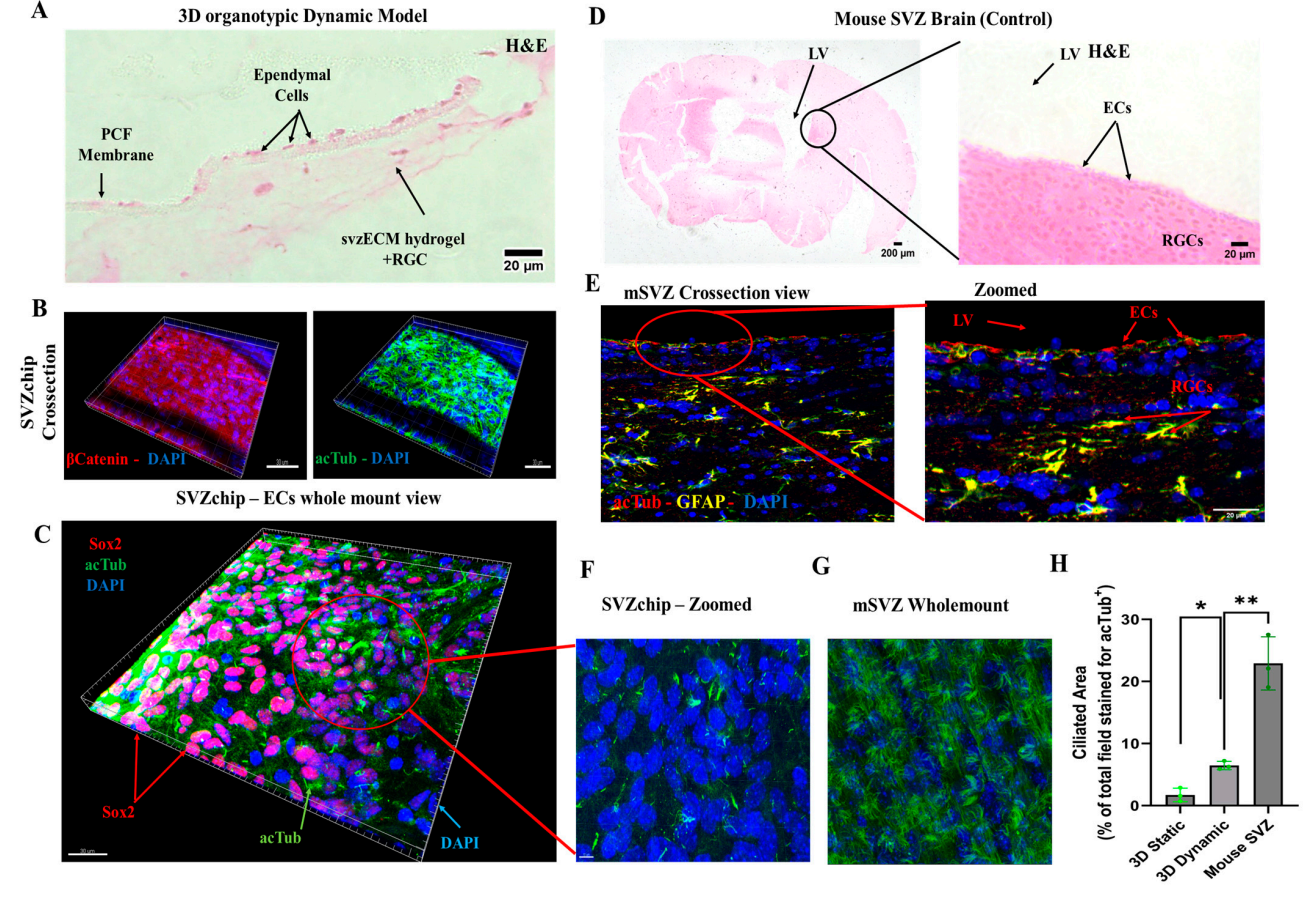
Dynamic in vitro 3D organotypic model of the SVZ. (**A**) H&E cross-section of the 3D organotypic model. It can be observed that the ependymal cells are attached to the porous PET membrane. On the side, the progenitor pRGCs are encapsulated in the svzECM. (**B**) Immunofluorescent images showing a cross-section of the whole sample, including the 3D svzECM hydrogel, the PET membrane, and the ECs stained against β-catenin, acTub, and DAPI. (**C**) Wholemount view of the bottom surface of the PET membrane showing the cilia generation throughout the dynamic sample, along with Sox2+ cells. (**D**) Histological images showing the mouse SVZ that was used as a native in vivo control. ECs can be seen on the epithelial layer of the VZ, while the adult population of pRGCs is below the ependymal epithelium. (**E**) Immunofluorescent staining of a cross-sectioned mouse SVZ stained against acTub in red, GFAP in yellow, and DAPI in blue. (**F**) Wholemount zoomed image of C, showing the cilia generated in the organotypic dynamic culture versus (**G**) the wholemount mouse SVZ that was used as the native control tissue sample. (**H**) Quantification of the ciliated area as a percentage of the total field stained for acTub^+^. Area-based analysis was performed using ImageJ on wholemount confocal z-stacks, calculating the total acTub^+^ signal per condition. This method was selected due to the dense, multicellular architecture of the samples and technical limitations that precluded the reliable segmentation of individual cilia tufts. This is an area-based quantification of the acTub^+^ signal, not individual cilium counting. Data are presented as the mean ± SEM, n = 3 (* *p* < 0.05, ** *p* ≤ 0.01). Panels (**B**,**C**,**F**) were generated using Imaris software, allowing for 3D volumetric reconstruction of the SVZonChip structure. These reconstructions enabled the characterization of hydrogel-embedded Sox2+ progenitors and acTub+ multiciliated cells in situ. This approach was chosen to visualize the full-tissue architecture rather than isolated 2D sections. For high-resolution images, please refer to the Supplementary Figures.

The physiological relevance of the 3D organotypic model was assessed using histological and immunohistochemical techniques. Cross-sectional images of the cell culture chamber (Figure 6A) showed the presence of ependymal cells (ECs) in the porous membranes in the microfluidic chip, while proliferating radial glial cells (RGCs) were encapsulated within the bovine svzECM matrix. The dimensions of the two chambers allowed the observation of cells at the edges throughout the culture period. Staining for β-catenin (Figure 6B, Figure S5 and Video S1) revealed the formation of an epithelial layer, with acTub marking the cilia on the surface of ECs, as confirmed by DAPI counterstaining. Three-dimensional-reconstructed wholemount images of the bottom layer of the membrane (Figure 6C, Figure S6 and Video S2) demonstrated a mature ependymal epithelium with numerous multiciliated cells. The growth of RGCs in the svzECM and ECs on the PET membrane confirmed the success of the cell seeding and culturing method. Three-dimensional fluorescence images further revealed an even distribution of all cell types across the membrane and hydrogel, forming a layered structure. The architectural and physiological similarities between the 3D organotypic model and the mouse lateral ventricle were evident upon comparison with histological images from the mouse brain (Figure 6D and Figure S4). Mouse SVZ cross-sections showed similar staining patterns for cilia in ECs (acetylated tubulin), GFAP-positive RGCs, and DAPI-stained nuclei, matching the organization in the SVZonChip. Immunostaining revealed that RGCs and ECs in the model exhibited comparable cellular arrangements, acTub expression, and cell–cell contact markers (β-catenin), which were evenly distributed, similar to the lateral ventricles of the mouse brain. The incorporation of svzECM created a microenvironment that enabled RGCs to adopt a phenotype resembling that of the SVZ, with the hydrogel providing mechanical support for cell growth (Figure 6B, Figure S5 and Video S3). Additionally, ECs were fully differentiated, forming cilia, as seen in zoomed images (Figure 6F and Figure S9), which closely resembled the cilia observed in wholemount images of the mouse SVZ stained for acTub.

To better visualize the entire architecture of the SVZonChip, including hydrogel-embedded cell populations, Figure 6B,C,F were generated using Imaris software (Imaris 9.1). This enabled 3D volumetric reconstruction, allowing identification of Sox2+ progenitors and acTub+ multiciliated ependymal cells in situ. This approach was chosen to present the full-tissue organization and cell distribution rather than isolated 2D sections, which are limited in the context of densely populated hydrogels.

Finally, the ciliated area (i.e., the percentage of the total field stained for acTub^+^) was quantified from confocal z-stacks using ImageJ. Quantification was based on the total acetylated tubulin (acTub) signal area, normalized against wholemount mouse SVZ tissue, which served as the in vivo reference for multiciliated ependymal epithelium. This method was selected due to the dense, multicellular architecture of wholemount samples and the technical limitations of segmenting individual cilia tufts in z-stack projections using ImageJ. While emerging tools such as *CiliaQ* [57] and *ACDC* [58] offer automated approaches to cilia quantification, they are optimized for distinct imaging contexts, such as monolayers or airway epithelia, and were not applied in this study. Our area-based approach allowed consistent quantification across conditions and reflected the biological benchmark of the native SVZ. An increase in the acTub+ cilia formation was observed in the dynamic over the static condition (*p* < 0.05 (*)) vs. the in the mouse in vivo wholemount SVZ (*p* ≤ 0.01 (**)) (Figure 6H).

## 4. Discussion

Understanding the physiology of the SVZ NSCN and its influence on NSC behavior requires in vitro models that closely mimic the physiological conditions of the NSCN. At present, there is a significant lack of advanced tools for effectively monitoring and screening the dynamic interactions within the SVZ NSCN. Most existing methods rely heavily on static cultures or animal models. While these approaches provide valuable insights, they fall short in replicating the complexities of the human NSCN microenvironment. This underscores the urgent need for robust organotypic platforms that not only recreate the anatomical and biochemical architecture of the SVZ but also incorporate its dynamic fluidic and mechanical characteristics. Developing such platforms could significantly advance neural tissue engineering (NTE) and regenerative medicine (RM) by enabling high-throughput screening of factors influencing neural stem and progenitor cell behavior. Additionally, these platforms would provide a more ethical and sustainable alternative to animal models by reducing reliance on animal usage, thus aligning with the 3Rs principle (replacement, reduction, and refinement). One major challenge in fabricating these platforms lies in the dependency on primary cells harvested from animals, which introduces variability and limits scalability. The heterogeneity of isolated primary radial glial cells (pRGCs), predominantly comprising neuronal and astroglial precursors, further complicates reproducibility. To address these limitations, there is a critical need to develop off-the-shelf cell sources, such as immortalized cell lines or induced pluripotent stem cell (iPSC)-derived neural cells, which can be standardized to generate organotypic cultures with consistent and reproducible properties. This would ensure accessibility and scalability for widespread research and drug discovery applications [59,60].

The subventricular zone (SVZ) niche is shaped by converging signals from the cerebrospinal fluid (CSF) and the vasculature, which jointly regulate neural stem cell (NSC) maintenance, proliferation, and lineage specification. Among these, fibroblast growth factor 1 (FGF1) and epidermal growth factor (EGF) are soluble cues secreted by the CSF and vascular endothelium, respectively, and have been shown to enhance NSC activation and neurogenic output [61,62,63]. Their complementary roles in sustaining neurogenesis and regulating quiescence are well-documented in both in vivo and in vitro models. In a related microfluidic study, Park et al. (2017) demonstrated that NSCs cultured under flow conditions with EGF and bFGF (FGF2) showed enhanced proliferation and radial glia-like morphology, highlighting the importance of integrating mechanical and biochemical cues [64].

In our SVZonChip platform, we incorporated EGF and FGF2 in the upper perfusion chamber to maintain pRGCs in a progenitor-like state under both static and dynamic co-culture conditions (see Section 2.6). While FGF1 was not included in the current study, its role in NSC quiescence and CSF-mediated signaling is well-recognized. Future chip iterations will explore the targeted inclusion of FGF1 using perfusion delivery to recapitulate spatiotemporal gradients and support context-specific NSC responses. Additionally, we are pursuing the integration of endothelial cell co-cultures to better simulate angiocrine signaling, shear stress responses, and neurovascular interactions within the SVZ niche. These refinements are expected to improve the architectural and functional fidelity of the model while also enabling more precise control over defined precursor pools for regenerative screening applications.

Expanding on this platform’s design, this study hypothesized that a tissue-specific decellularized SVZ extracellular matrix (svzECM) incorporating RGCs and ECs in a microphysiological system (MPS) could facilitate the development of an SVZonChip model capable of responding to environmental cues. This approach aimed to create a more physiologically relevant environment that closely mimics the native SVZ, allowing for better modeling of stem cell behavior and neurogenic potential.

Although the current study focused on early SVZ architecture using GFAP, Sox2, and acTub, we acknowledge that future experiments incorporating markers such as Ki67 and Dcx will be important for assessing active proliferation and neuroblast differentiation. These markers are currently included in follow-up studies aimed at functionally validating the neurogenic capacity of the SVZonChip model [6]. Ki67 and Dcx are both crucial for evaluating proliferative and neurogenic activities. Ki67 is a well-known marker of proliferating cells, indicating cell cycle activity and helping to identify actively dividing progenitors [65]. Dcx, a neuroblast-specific marker, is key for identifying the transition from progenitor cells to differentiating neuroblasts within the SVZ, a hallmark of neurogenesis [66]. Therefore, incorporating these markers will allow for a more complete validation of the SVZonChip’s functionality. To this end, while this study established a solid baseline by focusing on progenitor differentiation and ciliary activity, future work will focus on addressing these functional markers to further improve the model’s fidelity. These steps will be essential for demonstrating the model’s full neurogenic potential. Recent work has demonstrated that in 3D culture systems, cell–cell interactions and matrix properties complicate the resolution of proliferation and neurogenesis markers in dense tissues [65]. To address these challenges, bovine SVZ tissue was decellularized, and an svzECM hydrogel was developed to construct the SVZonChip model. This decellularized ECM provides a unique opportunity to retain the native microarchitecture and biochemical cues essential for NSC maintenance and differentiation. Developing region-specific ECM substrates from whole SVZ tissue represents a promising strategy for advancing NTE and RM. The results demonstrated the successful removal of cellular material during decellularization while preserving the svzECM microarchitecture, as shown by H&E staining (Figure 1B,C). This preservation is critical, as the structural integrity of the ECM influences cell adhesion, migration, and lineage specification within the hydrogel environment. Similar findings have been reported in previous studies using decellularization techniques [67,68]. Proteomic analysis of the decellularized svzECM identified expected ECM proteins, with collagen IV as the most abundant (13.2% of the total protein mass), followed by laminin (10.41%). Other ECM components, including neurocan (2.45%) and tenascin R (2.27%), were also detected (Figure 2A). While these proteins are known components of the svzECM [69,70], this study is, to the best of our knowledge, the first to quantify the components of the svzECM. A 20:80 mixture of the svzECM and commercial collagen type I was used to create a hydrogel suitable for 3D MPSs. Encapsulation of pRGCs in the svzECM hydrogel demonstrated excellent cell viability, proliferation (Figure 1E), and expression of progenitor markers such as GFAP and SOX2 (Figure 1F). This highlights the potential of the svzECM hydrogel to mimic the native stem cell niche, promoting cellular proliferation and maintenance of stemness markers. Incorporating collagen type I improved the mechanical and structural integrity of the svzECM hydrogel while preserving key ECM proteins critical for recapitulating the NSCN. Previous studies have also incorporated additional collagen I into decellularized ECM hydrogel scaffolds to enhance mechanical properties [71]. Ultrastructural characterization using SEM imaging revealed a uniform, staggered collagen fiber microstructure (Figure 4A–C), consistent with observations from earlier research [72,73]. This structured fiber alignment is crucial for providing the biomechanical cues necessary for cellular organization and migration within the hydrogel matrix. Rheological analysis confirmed the viscoelastic properties of the svzECM hydrogel, with G′ greater than G″ and a linear shear range of approximately 1.5% (Figure 4F). Mouse pRGCs isolated from lateral ventricles and cultured in a proliferation medium for two days retained their progenitor phenotype, as demonstrated by the expression of markers such as p73 and FOXJ1 (Figure 4B). Differentiation of pRGCs into mature ECs was also observed, with the acTub+ and p73− protein markers confirming successful maturation (Figure 4C). These findings suggest that the svzECM hydrogel not only maintains progenitor characteristics but also facilitates lineage progression towards mature ependymal cells.

The low expression levels of p73 and FoxJ1 observed in these early-stage cultures are consistent with the known temporal sequence of ependymal differentiation, where these transcription factors emerge more robustly during the later stages of commitment. These cells were only exposed to the differentiation medium for three days, whereas full ependymal maturation typically requires at least 15 days in vitro [53]. In contrast, the presence of acetylated tubulin-positive cilia at this early time point suggests that the initial steps of ciliogenesis were already underway in a subset of cells [74]. This early-stage experiment was designed to assess the baseline lineage responsiveness [75] of pRGCs and validate their suitability for downstream co-culture applications within the SVZonChip platform. Taken together, the observed marker profiles are consistent with early lineage commitment rather than terminal differentiation and support the intended use of these cells as biologically responsive precursors within the SVZonChip system.

This underscores the platform’s potential for applications in regenerative medicine, where controlled differentiation and lineage tracing are essential for therapeutic screening and disease modeling.

The svzECM hydrogel supported RGCs in both static and dynamic co-culture conditions. For static organotypic co-cultures, ECs generated cilia (acTub+), while pRGCs located on the upper portion of the co-culture expressed Sox2+, forming an epithelial-like barrier (Figure 4D–G) (Figures S4, S7 and S8), effectively mimicking the SVZ NSCN microarchitecture. This differentiation pattern demonstrates the capability of the hydrogel to sustain progenitor populations and drive differentiation in a manner reflective of the native SVZ environment. In dynamic conditions, a microfluidic device with a two-layer insert was employed to replicate the ependymal and hypocellular layers of the SVZ. The lower chamber received a constant flow of differentiation medium to simulate cerebrospinal fluid (CSF) dynamics, an essential mechanical cue for maintaining SVZ homeostasis. ECs possess motile cilia and are influenced by the mechanical forces of the CSF [76,77]. In this study, the Kirkstall Quasi Vivo (QV600) interconnected chamber system was employed, enabling 15-day co-culture without medium leakage and ensuring continuous differentiation and cell survival under moderate flow (1 µL/mL) consistent with in vivo CSF flow rates [78,79,80,81,82]. Histological and immunohistochemical analyses confirmed the proper formation of SVZ-mimicking layers. ECs in the lower chamber were stained with acTub+, indicating cilia formation, while RGCs encapsulated in the svzECM expressed GFAP and Sox2+, confirming their progenitor phenotype (Figure 6B,C,F). The dynamic model showed enhanced physiological relevance, with more acTub+ ECs compared to static conditions (Figure 6H). This suggests that shear stress from CSF-like flow enhances cilia production. This enhancement is indicative of the role of shear stress in promoting ciliogenesis and cellular organization, suggesting that flow-based culture conditions are instrumental in achieving more in vivo-like phenotypes.

While the epithelial organization of the upper chamber population was only partially observed, we have occasionally noted rosette-like cellular arrangements in prior experiments using this platform, which resemble the early ependymal layer formations described in the literature [54]. Such features suggest that the membrane-adherent ependymal-like cells (ECs) are beginning to organize and polarize, potentially reflecting an initial step toward epithelial differentiation. However, the variability observed may be attributed to the developmental stage of differentiation or specific culture conditions. Future studies will focus on a more detailed analysis of epithelial maturation and ciliary complexity by incorporating additional markers such as N-cadherin, which identifies adherens junctions, and γ-tubulin, which localizes to basal bodies, anchoring motile cilia [6]. Additionally, we are planning transepithelial electrical resistance (TEER) measurements to functionally assess barrier integrity and epithelial cohesion over time, which will help refine the SVZonChip model and better capture the full dynamics of the SVZ niche.

To capture the complex spatial organization of the hydrogel-embedded cell population, we employed 3D volumetric reconstruction using Imaris software (Figure 6B,C,F). This approach enabled THE visualization of Sox2+ progenitor cells and acetylated tubulin (AcTub+) multiciliated ependymal cells within the full 3D context of the SVZonChip. Although single-plane characterization was limited by the density and opacity of the ECM, wholemount imaging and advanced reconstruction enabled us to assess cell distribution across the hydrogel. We are actively optimizing protocols for deeper immunolabeling and clearing to improve cellular profiling within the 3D compartment.

While our analysis focused on quantifying the total acetylated tubulin (acTub) signal area as a proxy for multiciliation, this approach was chosen due to the density of the wholemount samples and technical limitations in segmenting individual cilia across z-stacks. This method aligns with the broader use of global acTub signal as a tissue-level indicator of ciliation, especially in neuroepithelial or multicellular contexts where individual cilium counting is not practical. Automated tools such as *CiliaQ* [57] and *ACDC* [58] support this rationale, although they were not applied here due to their optimization for distinct imaging setups. The svzECM hydrogel provided a supportive microenvironment that allowed RGCs to adopt an SVZ-like phenotype, closely resembling the native NSCN. Despite challenges, including the heterogeneity of isolated pRGC populations, the proposed model successfully replicated the distinct layers of the SVZ. Future work will aim to refine differentiation protocols to increase the proportion of acTub+ ECs and further characterize RGC populations. Ultimately, the development of organotypic SVZonChip platforms using off-the-shelf cell sources could revolutionize neural research by providing a scalable, reproducible, and ethical alternative to animal models. This study represents a significant step forward, demonstrating the feasibility and physiological relevance of an SVZonChip model that captures both the static and dynamic aspects of the NSCN microenvironment.

## 5. Conclusions

Beyond its immediate applications in understanding the SVZ microenvironment, the SVZonChip model holds promise for translational research and therapeutic screening. By incorporating patient-derived iPSCs or genetically modified cell lines, this platform could be tailored to study disease-specific alterations in the NSCN, such as those observed in neurodegenerative diseases or brain tumors. Moreover, the dynamic fluidic capabilities of the model provide a unique opportunity to explore how mechanical forces, such as shear stress from CSF flow, influence cell behavior, lineage commitment, and regenerative capacity. These insights could pave the way for novel therapeutic strategies, such as modulating mechanical cues to enhance endogenous repair mechanisms or delivering targeted treatments via the CSF. Additionally, the proposed platform’s adaptability offers significant potential for investigating other tissues where dynamic conditions, such as fluid flow, shear stress, or cyclic mechanical forces, are crucial and necessary for maintaining physiological function, such as vascular, cardiac, or musculoskeletal tissues. The versatility of the SVZonChip system also lends itself to drug screening and toxicity assays, offering a human-relevant alternative for evaluating the efficacy and safety of neuropharmaceuticals, thereby accelerating the path from bench to bedside. Furthermore, the SVZonChip platform is compatible with a range of stem cell sources beyond iPSCs, including mesenchymal stem cells, such as adipose-derived stem cells (ADSCs), and established neural stem cell lines. This versatility enables researchers to tailor the system to diverse experimental objectives, whether focused on disease modeling, regenerative neurobiology, or high-throughput drug screening. By accommodating distinct stem cell populations, the SVZonChip expands its translational potential across multiple biomedical applications.

## Data Availability

The original contributions presented in the study are included in the article/Supplementary Material, further inquiries can be directed to the corresponding author.

## Supplementary Materials

The following supporting information can be downloaded at: https://www.mdpi.com/article/10.3390/bioengineering12060562/s1, 1. Video S1: Establishment of the organotypic co-culture after 7 days in vitro, visualized through whole-mount imaging of the membrane’s underside, 2. Video S2: Whole-mount images of the upper side of the membrane demon-strate the formation of the hydrogel with embedded RGCs, marked by β-Catenin expression, 3. Video S3: Dynamic in vitro 3D Organotypic Model of the SVZ.
