# Supplementary material for: A 3D SVZonChip Model for In Vitro Mimicry of the Subventricular Zone Neural Stem Cell Niche
Source: Bioengineering (Basel). 2025 May 23;12(6):562. doi: 10.3390/bioengineering12060562 (PMC12189603; doi:10.3390/bioengineering12060562)
Supplement: Supplementary file 1 [file bioengineering-12-00562-s001.zip › Supplemented Figures.pdf]

Supplemented Data

A 3D SVZonChip Model for In Vitro Mimicry of the Subventricular Zone Neural Stem Cell Niche

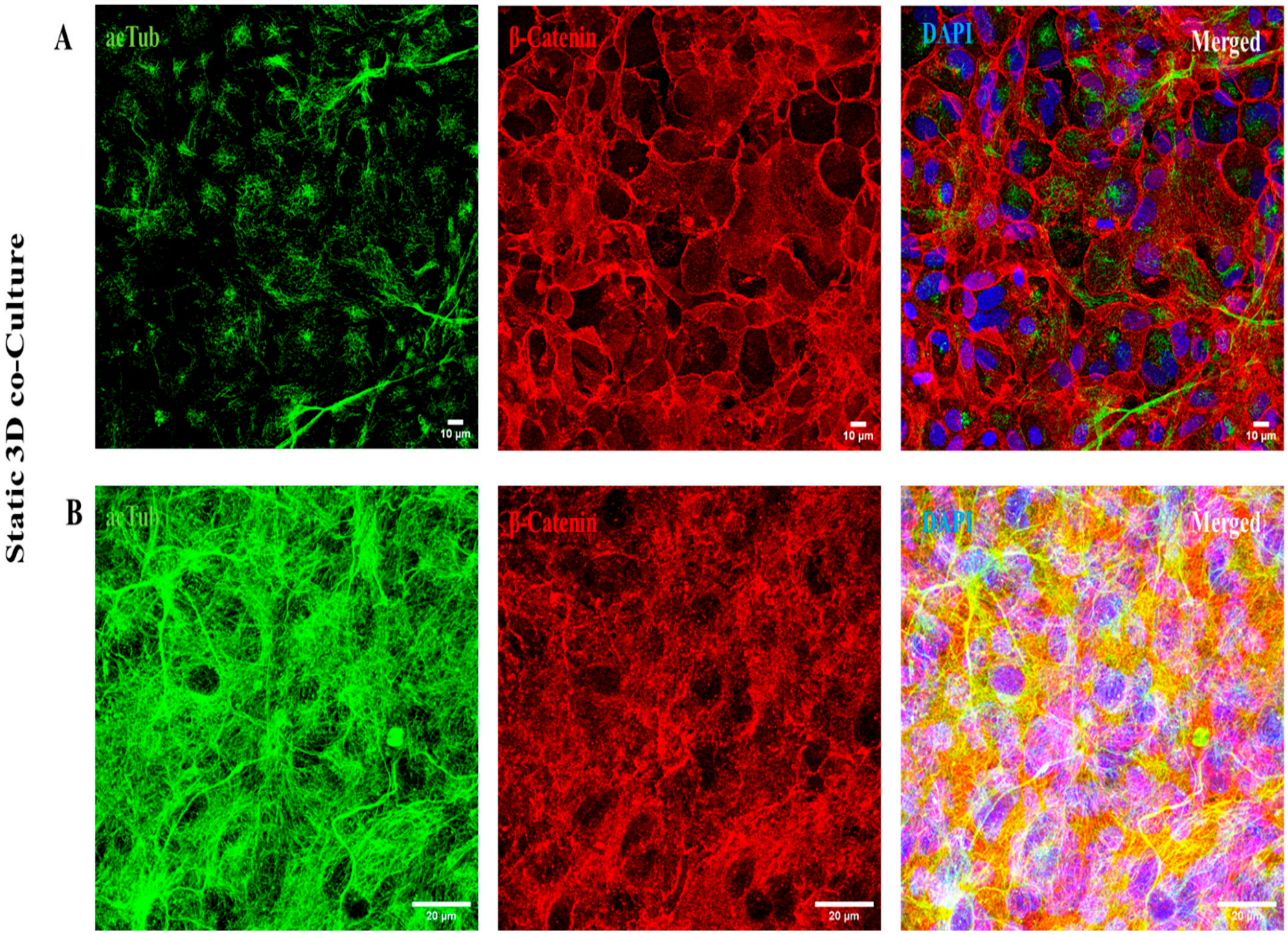

<u>Figure S1:</u> (A) Establishment of the organotypic co-culture after 7 days *in vitro*, visualized through whole-mount imaging of the membrane's underside. The epithelial barrier is clearly delineated by β-Catenin expression, while the presence of motile cilia is highlighted with acTub staining (green). (B). The signal appears more diffuse and less defined compared to the structured epithelial barrier observed in panel (A), reflecting the three-dimensional arrangement and cellular interactions within the hydrogel matrix.

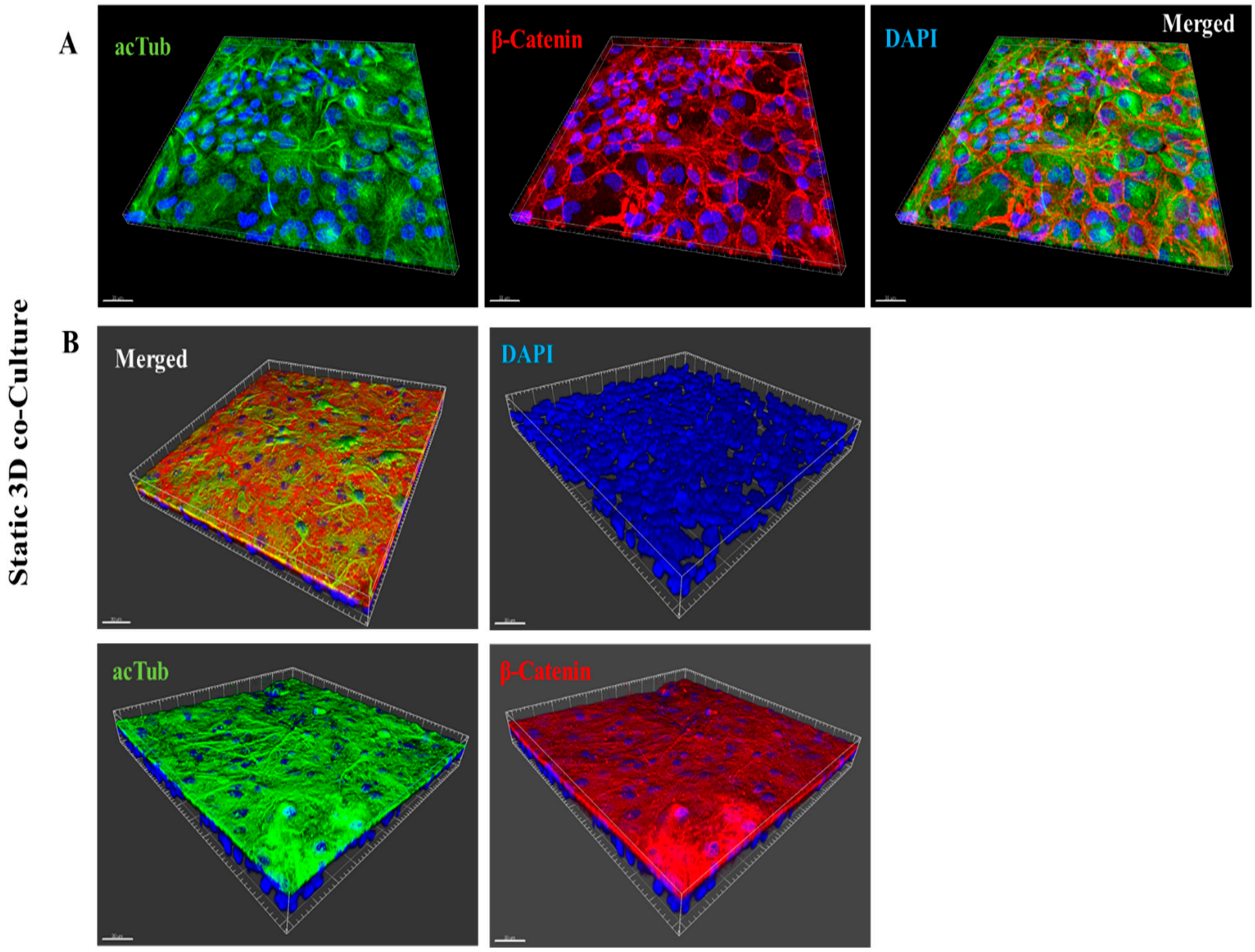

<u>Figure S2</u>: 3D reconstruction images of supplemented figure 1 showing the characteristic epithelial barrier from different perspective (A) Whole-mount imaging of the membrane's underside. (B) Whole-mount 3D reconstruction immunofluorescence images of the 3D svzECM hydrogel, and the ependymal cells (ECs). The structures are labeled with β-Catenin (epithelial barrier), acTub (cilia), and DAPI (nuclei), with the visualization performed using Imaris software to achieve detailed spatial representation.

A

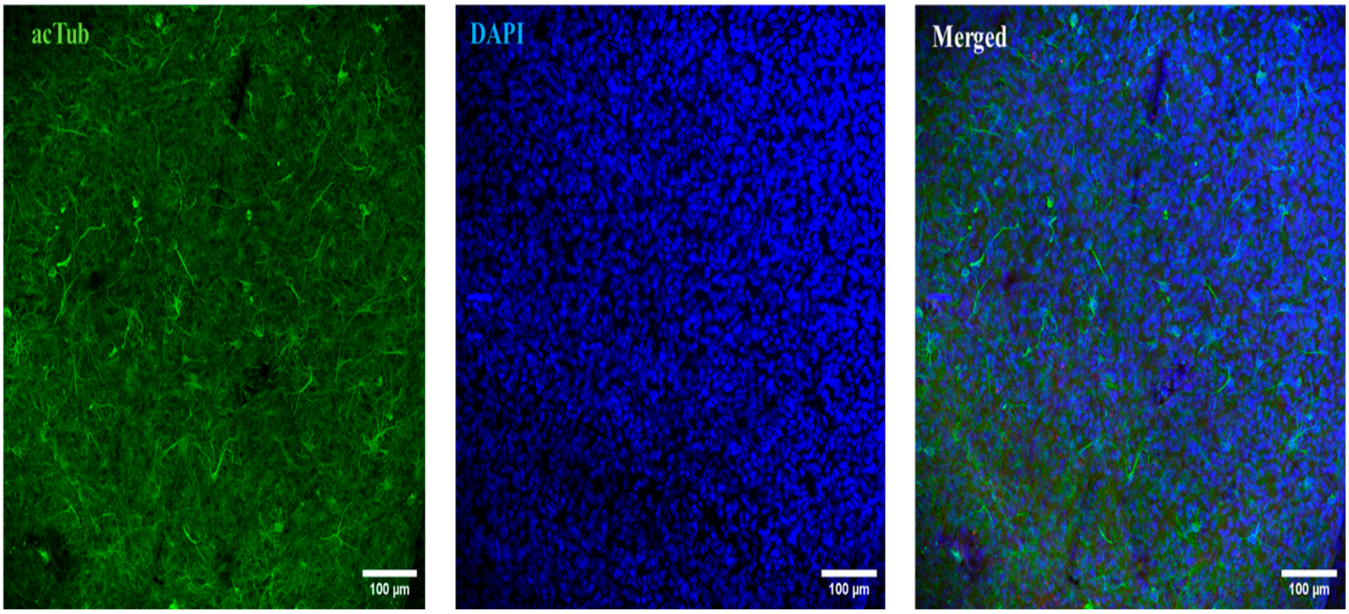

<u>Figure S3:</u> (A) Immunofluorescence images of the static organotypic co-culture, represented by whole-mount images captured from the top view of the hydrogel. The images highlight the diversity of cell types, including neural-like cells, as evidenced by their characteristic cell morphology.

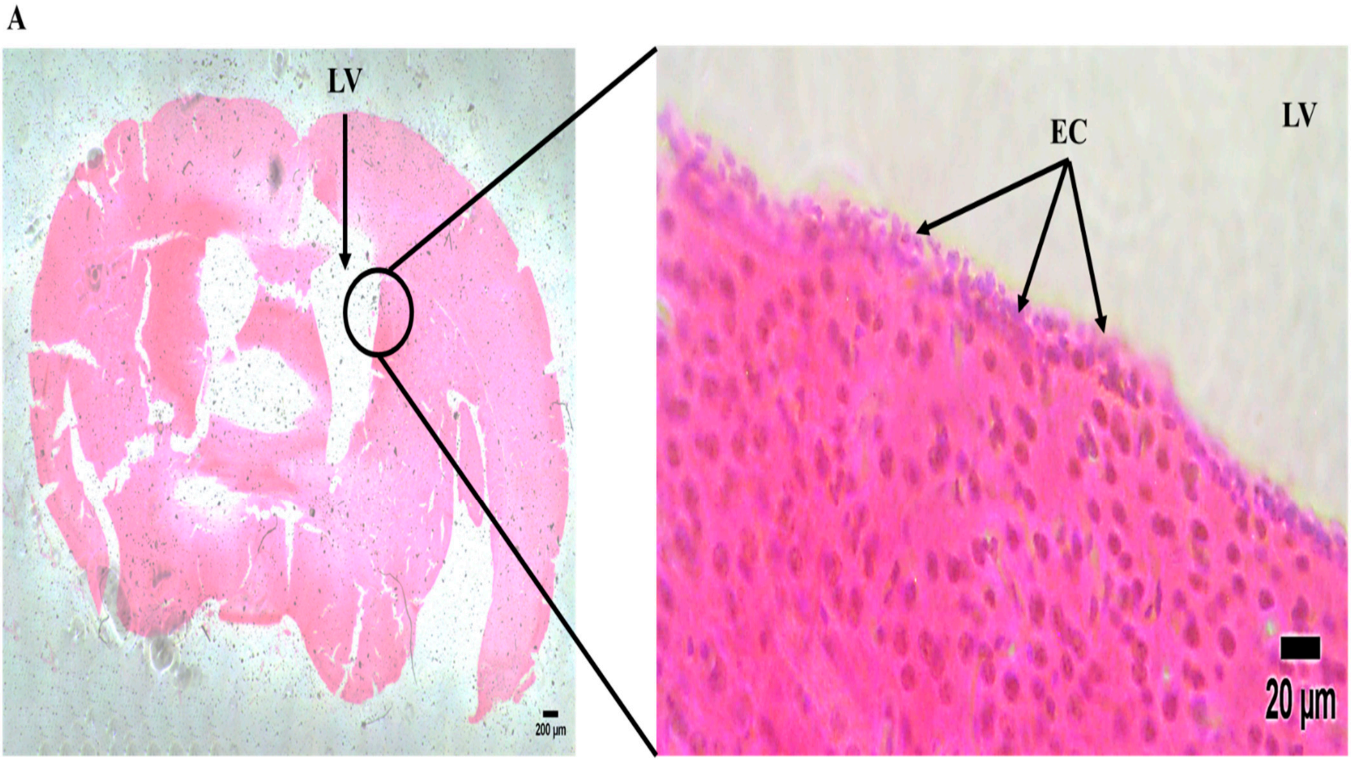

<u>Figure S4:</u> (A) Representative histological sections illustrating the mouse SVZ, which served as the native *in vivo* control. Ependymal cells (ECs) are visible in the epithelial layer of the ventricular zone (VZ), with the adult population of postnatal radial glia-like cells (pRGCs) positioned beneath the ependymal lining.

A

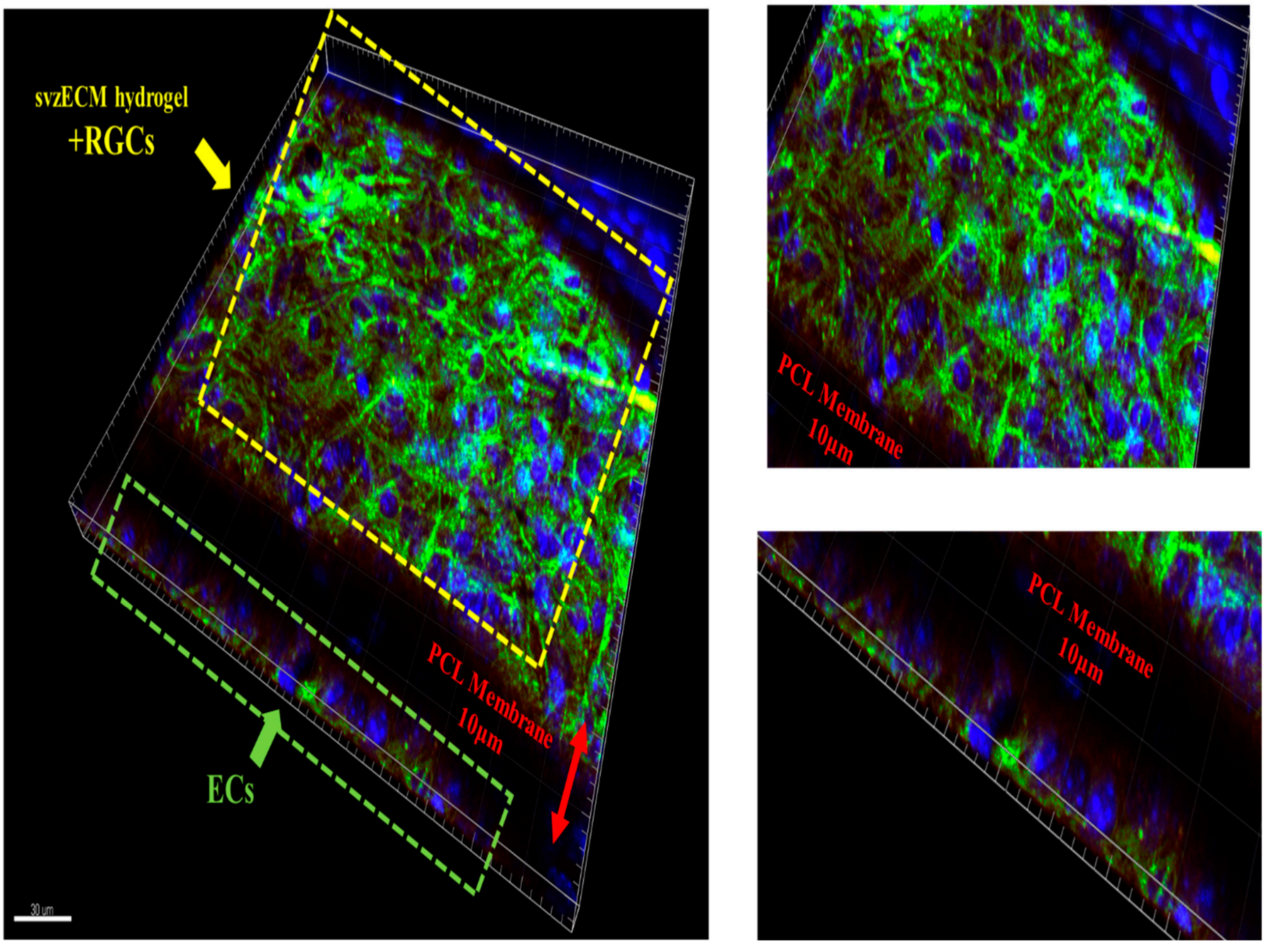

<u>Figure S5</u>: Dynamic in vitro 3D Organotypic Model of the SVZ. (A) 3D reconstruction of the whole-mount visualization of the PET membrane's underside, depicting the epithelial barrier formed beneath the 10 µm-thick PCL membrane. Cilia formation is clearly visible throughout the dynamic culture. Above the PCL membrane, a dense cellular network is observed within the svzECM hydrogel, illustrating the complexity and organization of the model.

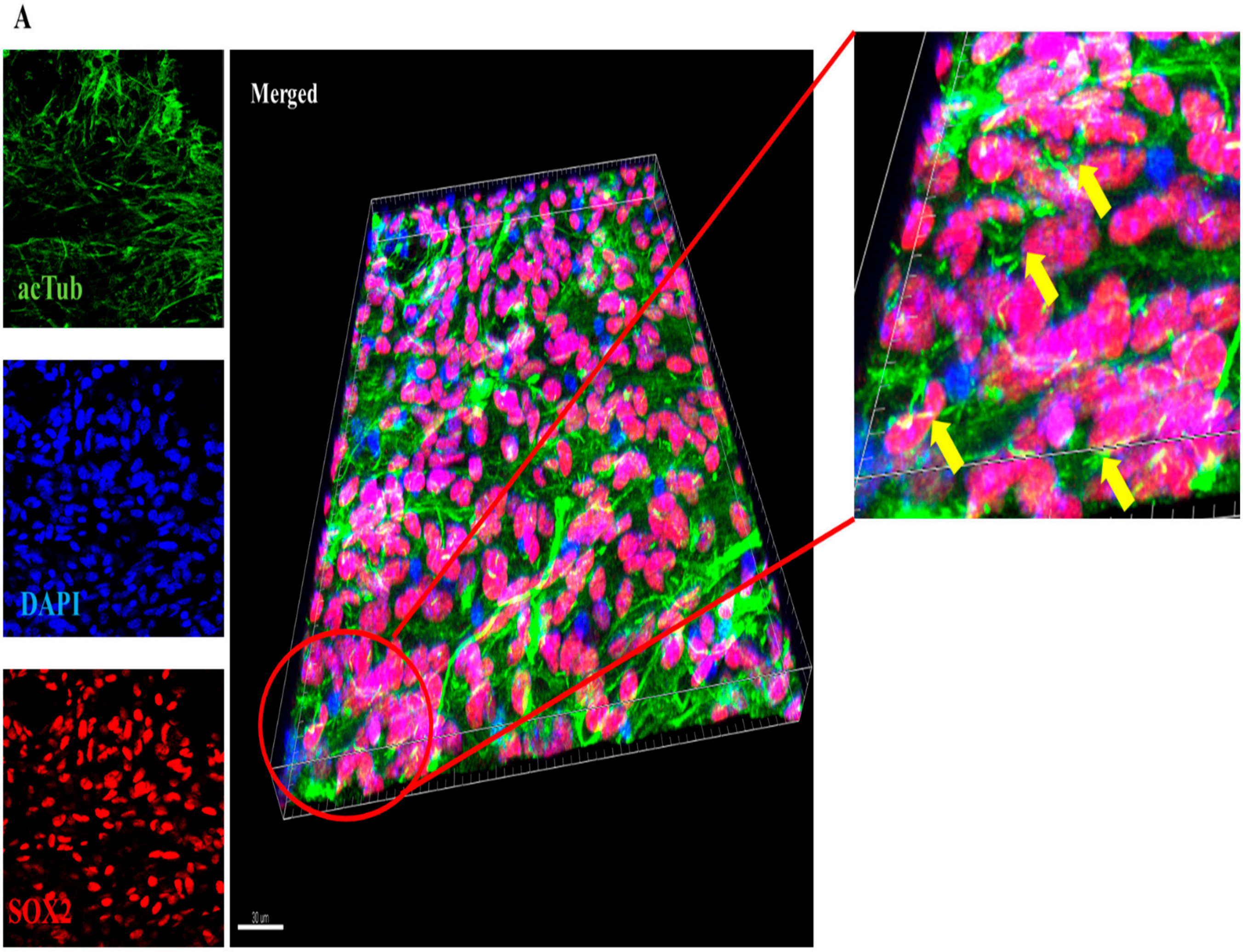

<u>Figure S6:</u> Dynamic *in vitro* 3D organotypic Model of SVZ. (A) Whole-mount visualization of the PET membrane's underside, demonstrating cilia formation across the dynamic culture along with the presence of Sox2+ cells.

A

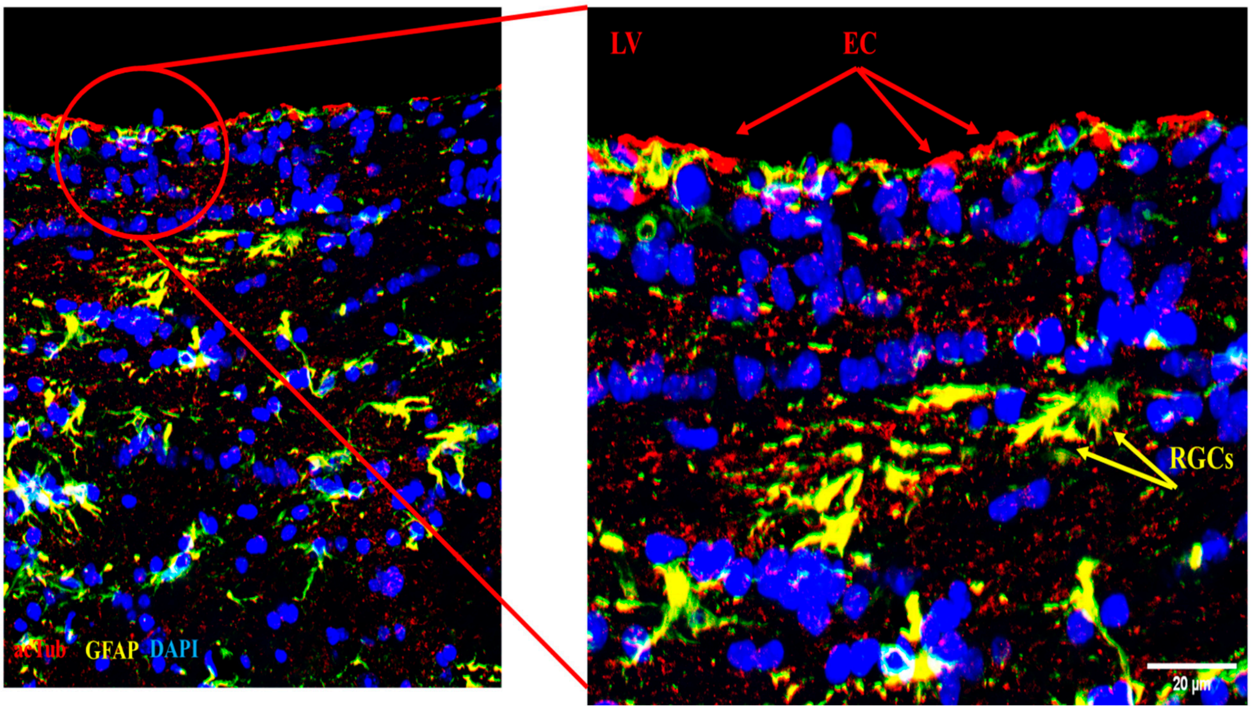

<u>Figure S7:</u> (A) Immunofluorescence staining of a cross-sectioned mouse SVZ, labeled with acTub in red, GFAP in yellow, and DAPI in blue.

A

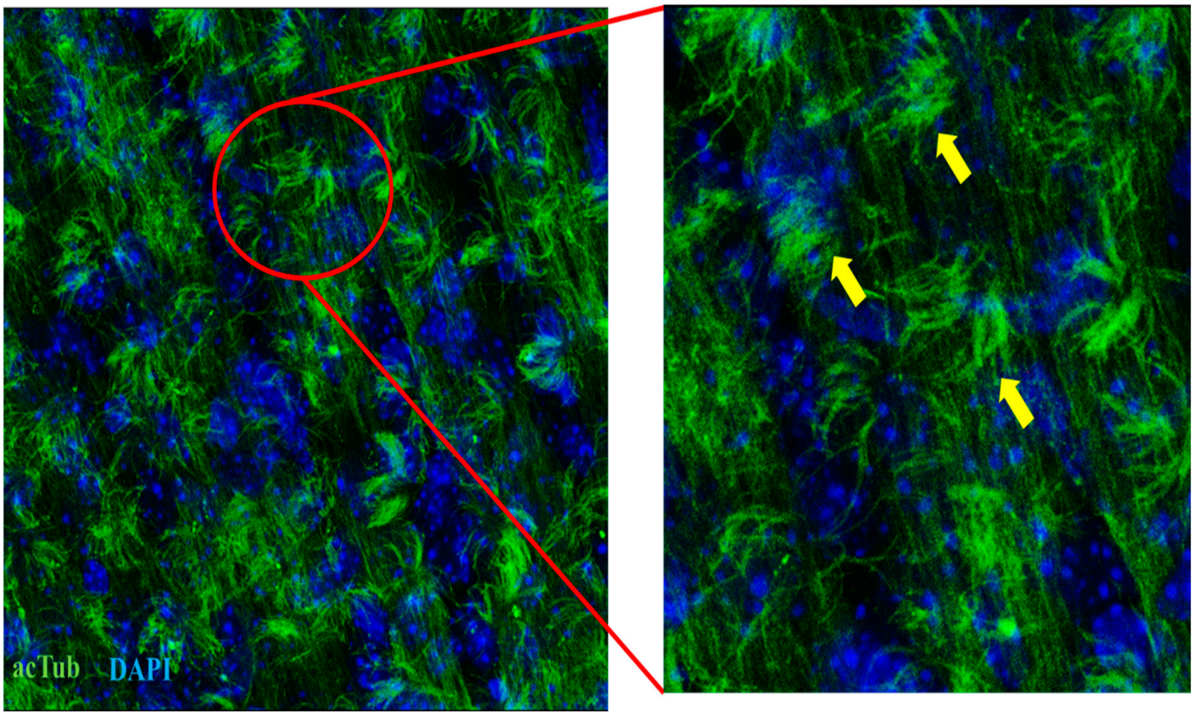

Figure S8: (A) Whole-mount preparation of the mouse SVZ, utilized as the native control tissue sample.

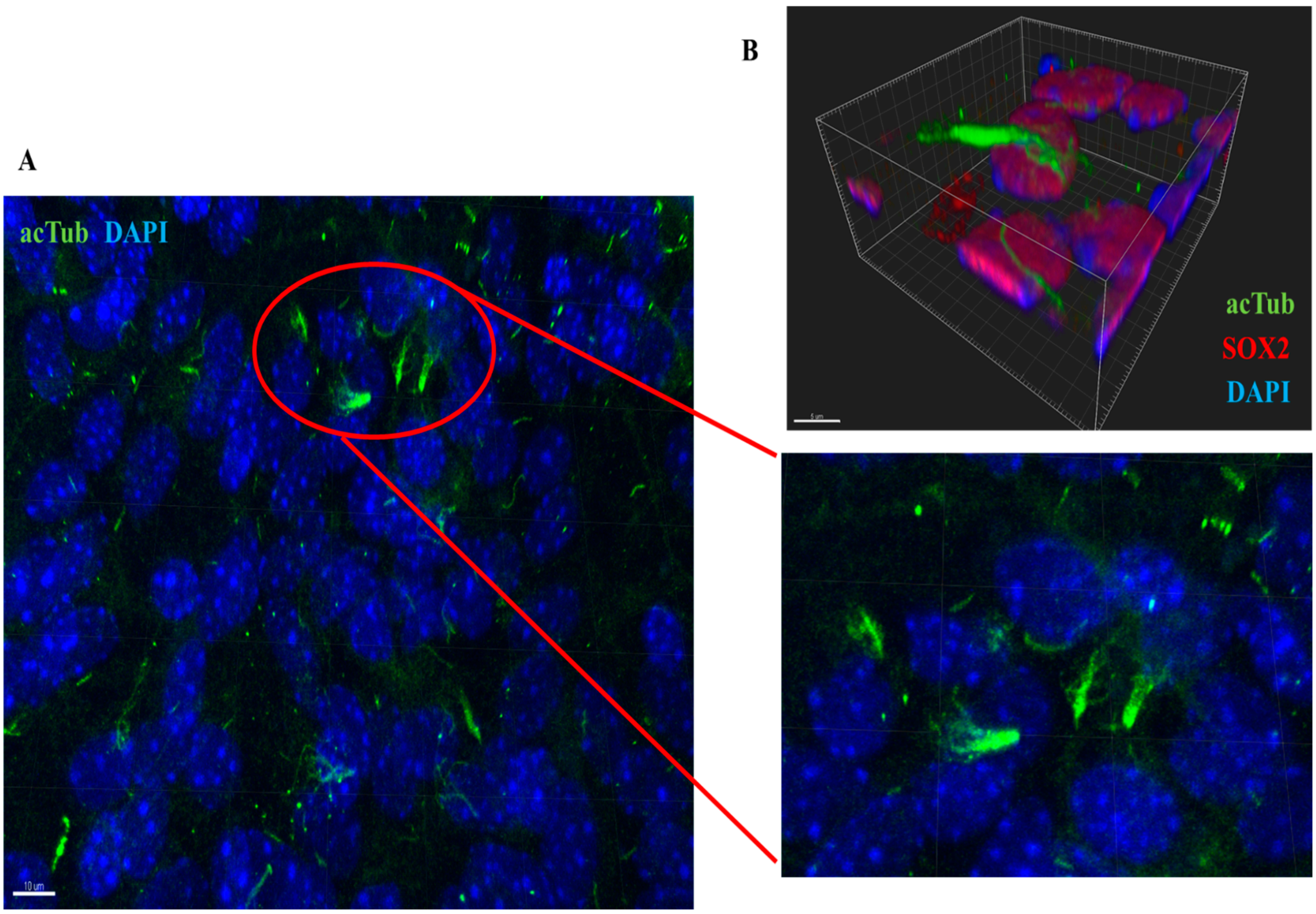

<u>Figure S9:</u> **(A) Magnified view of the whole-mount image from Figure 6C, highlighting the cilia generated within the organotypic dynamic culture. (B) 3D reconstruction of a close-up image of ependymal cells located on the ependymal membrane. The whole-mount immunofluorescence image showcases the ependymal barrier beneath the membrane, labeled with SOX2 (progenitor marker), acTub (cilia marker), and DAPI (nuclear stain), providing a detailed visualization of cellular organization.**
